# TLR3 Ligand Poly(I:C) Exerts Distinct Actions in Synovial Fibroblasts When Delivered by Extracellular Vesicles

**DOI:** 10.3389/fimmu.2018.00028

**Published:** 2018-01-29

**Authors:** Mojca Frank-Bertoncelj, David S. Pisetsky, Christoph Kolling, Beat A. Michel, Renate E. Gay, Astrid Jüngel, Steffen Gay

**Affiliations:** ^1^Center of Experimental Rheumatology, Department of Rheumatology, University Hospital Zurich, Schlieren, Switzerland; ^2^Department of Medicine, Duke University Medical Center, Durham, NC, United States; ^3^Medical Research Service, Durham VA Medical Center (VHA), Durham, NC, United States; ^4^Schulthess Clinic, Zurich, Switzerland

**Keywords:** double-stranded RNA, extracellular vesicles, Poly(I:C), synovial fibroblasts, inflammation, apoptosis, innate immunity

## Abstract

Extracellular vesicles (EV) can modulate the responses of cells to toll-like receptor (TLR) ligation; conversely, TLR ligands such as double-stranded RNA (dsRNA) can enhance the release of EV and influence of the composition and functions of EV cargos. Inflamed synovial joints in rheumatoid arthritis (RA) are rich in EV and extracellular RNA; besides, RNA released from necrotic synovial fluid cells can activate the TLR3 signaling in synovial fibroblasts (SFs) from patients with RA. Since EV occur prominently in synovial joints in RA and may contribute to the pathogenesis, we questioned whether EV can interact with dsRNA, a TLR3 ligand, and modify its actions in arthritis. We have used as model the effects on RA SFs, of EV released from monocyte U937 cells and peripheral blood mononuclear cells upon stimulation with Poly(I:C), a synthetic analog of dsRNA. We show that EV released from unstimulated cells and Poly(I:C)-stimulated U937 cells [Poly(I:C) EV] differ in size but bind similar amounts of Annexin V and express comparable levels of MAC-1, the receptor for dsRNA, on the vesicular membranes. Specifically, Poly(I:C) EV contain or associate with Poly(I:C) and at least partially protect Poly(I:C) from RNAse III degradation. Poly(I:C) EV shuttle Poly(I:C) to SFs and reproduce the proinflammatory and antiviral gene responses of SFs to direct stimulation with Poly(I:C). Poly(I:C) EV, however, halt the death receptor-induced apoptosis in SFs, thereby inverting the proapoptotic nature of Poly(I:C). These prosurvival effects sharply contrast with the high toxicity of cationic liposome-delivered Poly(I:C) and may reflect the route of Poly(I:C) delivery *via* EV or the fine-tuning of Poly(I:C) actions by molecular cargo in EV. The demonstration that EV may safeguard extracellular dsRNA and allow dsRNA to exert antiapoptotic effects on SFs highlights the potential of EV to amplify the pathogenicity of dsRNA in arthritis beyond inflammation (by concurrently enhancing the expansion of the invasive synovial stroma).

## Introduction

Extracellular vesicles (EV) are a heterogeneous group of vesicles that are secreted from cells to enter the extracellular space where they can exhibit a variety of immunological properties. EV vary in size, mechanisms of biogenesis, and molecular composition of vesicular membranes and intravesicular cargos, including a variety of lipids, proteins, and nucleic acids (1). The structure of EV can stabilize of vesicular cargos and thereby allows EV to act in intercellular communication and the transfer of informational molecules *in vivo* over varying distances (2). EV control fundamental cellular functions such as cellular migration, invasion, and immune responses (2, 3) and have a prominent role in human diseases, functioning as drivers of disease, disease biomarkers, or potential therapeutics (2, 3).

EV are increasingly recognized for their potentially important roles in the pathogenesis of autoimmune diseases, including rheumatoid arthritis [RA] (3, 4). Synovial fluid from patients with RA contains increased amounts of EV derived from platelets, monocytes, lymphocytes and neutrophils (5–8). These EV can promote the matrix-degrading and/or proinflammatory properties in synovial fibroblasts (SFs) and/or neutrophils (5, 6, 9, 10), thereby aggravating the arthritis; EV, however, can have also chondroprotective (7) and proresolving (11) functions, which could be exploited therapeutically (4). EV contain or can associate with proinflammatory cytokines (5), damage-associated molecular patterns (DAMPs) (11), or citrullinated autoantigens (6, 12) and can modulate their proinflammatory actions (6, 13) in inflammatory arthritis.

Signaling *via* toll-like receptors (TLRs) is central to the innate immune responses and the pathogenesis of autoimmune diseases, including RA (14). TLR ligands such as polyinosinic-polycytidylic acid [Poly(I:C)] can enhance the release of EV from a diversity of cell types that populate the synovium in RA (9) and can specify the composition and function of EV cargos (15). Conversely, EV can influence the responses of cells to TLR ligation (16). Poly(I:C) is a synthetic analog of double-stranded RNA (dsRNA) that activates the signaling *via* TLR3 and cytoplasmic dsRNA sensors such as melanocyte differentiation-associated 5 (MDA5, also known as IFIH1) (17, 18). Poly(l:C) is used to model the actions of extracellular dsRNA. Extracellular dsRNA is released *in vivo* from injured tissues and dying cells and can aggravate tissue damage *via* TLR3-dependent mechanisms (19). Synovial tissues from patients with RA contain increased amounts of extracellular RNA (20) and are rich in the expression of TLR3 (21). RNA released from necrotic synovial fluid cells activates the proinflammatory signaling *via* TLR3 in SFs, the key effector cells in joint inflammation and destruction in RA (22). Besides, stimulation of SFs with Poly(I:C) recapitulates the cytokine composition of synovial fluid in RA (23).

Since EV and extracellular RNA share the common extracellular space in inflamed synovial joints of patients with RA, they may interact to influence the functions of each other, thereby contributing distinctly to the activation of SFs and the pathogenesis of inflammatory arthritis. Here, we show that EV released from monocyte U937 cells stimulated with Poly(I:C) incorporate Poly(I:C) into their structure, at least partially protect Poly(I:C) from RNAse degradation and shuttle Poly(I:C) to SFs from patients with RA. While Poly(I:C)-containing EV could recapitulate the antiviral and proinflammatory effects of Poly(I:C) in RA SFs, these EV inverted the proapoptotic actions of Poly(I:C), thereby protecting SFs from death receptor-induced apoptosis. In sum, these findings suggest that the EV-rich milieu of inflamed synovial joints in RA fosters the pathogenicity of dsRNA by guarding the integrity of dsRNA and diversifying its actions beyond the effects of free dsRNA.

## Materials and Methods

### Cell Culture

Human U937 cells (Leibniz Institute DSMZ-German Collection of Microorganisms and Cell Cultures) were maintained in RPMI 1640 cell culture medium (Gibco by Life Technologies) supplemented with 10% fetal calf serum (FCS). U937 cells of passages 8–40 were used in the experiments. Human SFs were derived from synovial tissues of RA patients obtained during joint replacement surgery at the Schulthess Clinic, Zurich, Switzerland. All patients fulfilled the American College of Rheumatology 1987 criteria for RA (24). SFs were cultured in Dulbecco’s modified Eagle’s medium (DMEM, Gibco by Life Technologies) supplemented with 10% FCS and SFs of passages 4–8 were used in the experiments. For preparation of the cell culture media, FCS was heat inactivated at 56°C and was sterile filtered (0.22 µm). Cell cultures were negative for mycoplasma contamination as assessed by MycoAlert mycoplasma detection kit (Lonza).

Peripheral blood mononuclear cells (PBMCs) were isolated from peripheral blood of healthy donors (*n* = 3) by density gradient centrifugation using Ficoll-Paque™ according to manufacturer’s recommendations (Milteny Biotec). PBMCs were maintained in RPMI 1640 supplemented with 10% FCS in the presence or absence of Poly(I:C) for 16 h. For flow cytometry measurements of MAC-1 expression PBMCs were isolated from healthy donors using also the Ficoll-Percoll method as described in previous studies (25, 26).

### Treatment of U937 Cells and PBMCs and Preparation of EV

U937 cells and PBMCs were stimulated with 20 µg/ml high-molecular-weight Poly(I:C) [Poly(I:C) HMW, InvivoGen] for 16 h or were left untreated. EV were isolated from the conditioned media of 15 million U937 cells using differential centrifugation protocol. Cells were pelleted at 360 *g*, 6 min, RT, and the supernatants were centrifuged at 1,400 *g*, 10 min, RT to remove cell debris. Cell- and cell debris-free supernatants (~8 ml) were then centrifuged at 20,000 *g*, 4°C, 20 min to obtain EV pellets. EV pellets were washed in two subsequent centrifugation steps (20,000 *g*, 4°C, 20 min) with Dulbecco’s phosphate-buffered saline (DPBS, Gibco by Life Technologies) and finally resuspended in DMEM supplemented with 0.5% FCS. The supernatant from the last washing step of EV pellets (control Sup) served as a negative control for a potential carryover of residual soluble Poly(I:C) or soluble U937 cell-derived mediators *via* EV suspensions. Additionally, sterile filtered (220 nm filter) RPMI 1640 medium containing 10% FCS was incubated with 20 µg/mL Poly(I:C) for 16 h under the same conditions as cells. This medium was differentially centrifuged as described above to pellet potential residual FCS-derived EV (remaining after sterile filtering the cell culture media through 0.22 µm filters, a standard step during cell culture media preparation in our laboratory). FCS EV control samples were used in stimulation experiments to determine whether potential residual FCS-derived EV are responsible for the observed effects of Poly(I:C) EV. In a subset of experiments 15 million U937 cells were incubated with 100 ng/ml LPS from Escherichia coli J5 (List Biological Laboratories, # 301) or 10 ng/ml of recombinant human TNF alpha (R&D Systems, # 210-TA) and LPS EV or TNF EV, respectively, were isolated according to the protocol described above. The control Sup obtained from the last washing step of LPS EV pellets increased the expression of *IL-6* mRNA in SFs, pointing toward the potential LPS contamination in control Sup and LPS EV. Thus, we used only TNF EV in further analyses.

### Characterization of EV

Size distribution and number of EV were determined by nanoparticle tracking analysis (NTA) using NanoSight LM10 Instrument (NanoSight Ltd., number of tracks 322–772, temperature 28.8–28.9°C, viscosity 0.82 cP, 30 frames/s, time 60 s). NTA enables automatic tracking and sizing of particles based on their Brownian motion and diffusion coefficient, thereby measuring an absolute concentration of particles and the frequency distribution of particle sizes. For NTA, EV pellets derived from 15 × 10^6^ U937 cells (*n* = 2 per condition) were resuspended in sterile filtered DPBS and diluted 1:10. Undiluted FCS EV samples showed minimal contamination with particles, accounting for ~1% of all particles in media conditioned with cells.

In addition to NTA, EV were analyzed on FACSCanto II or FACSCalibur flow cytometers (BD Biosciences) using BD FACSDIVA or FlowJo V10, respectively. The settings for EV analysis were established with the Megamix fluorescent beads (Biocytex) according to the manufacturer’s protocol. EV were labeled with PE Mouse Anti-Human CD11b/Mac-1 (BD Pharmingen, #555388, 2.5 µg/ml), PE Mouse IgG1 κ Isotype control (BD Pharmingen, #555749, 2.5 µg/ml) or FITC-Annexin V (5 µl per sample, BD Pharmingen, 556419) for 15 min at RT in the dark, thoroughly washed and analyzed by flow cytometry.

The total amount of protein in EV pellets derived from 15 million U937 cells (lysed in 50 µl RIPA buffer) was determined spectrophotometrically using the Pierce™ BCA Protein Assay Kit (Thermo Fisher Scientific) according to the manufacturer’s instructions.

### Treatment of SFs

During coculture experiments with EV, SFs were maintained in DMEM supplemented with 0.5% FCS. 100,000 SFs were stimulated with EV derived from 3 × 10^6^ untreated or Poly(I:C)-stimulated U937 cells for 24 h. A set of negative control experiments included treatment of SFs with control Sup and FCS EV controls. SFs were directly stimulated with HMW Poly(I:C) (20 pg/ml to 20 μg/ml, InvivoGen, # tlrl-pic-5) or were transfected with HMW Poly(I:C) (0.02–1 µg/ml, InvivoGen, # tlrl-pic-5) using Lipofectamine 2000 (Invitrogen) according to manufacturer’s instructions. Apoptosis was induced in SFs with 200 ng/ml of recombinant human TNF-related apoptosis-inducing ligand (TRAIL) (R&D Systems, #375-TEC) or with 100 ng/ml human recombinant Fas ligand (FasL) (R&D Systems, #126-FL) in the absence or presence of Poly(:C) EV, Con EV, TNF EV or Sup controls. SFs were treated with TRAIL and directly stimulated with HMW Poly(I:C) (20pg/ml-20μg/ml, InvivoGen, # tlrl-pic-5) or transfected with HMW Poly(I:C) (0.02–1 µg/ml, InvivoGen, # tlrl-pic-5). To inhibit the NF-kB signaling, sc-514 (50 µM, EMD Millipore) was diluted in DMSO (Sigma) and added to the cell culture medium 1 h before treatment of SFs with EV in the presence or absence of TRAIL. DMSO, containing no sc-514 was used as a control. The efficacy of sc-514 (50 µM, EMD Millipore) in inhibiting the NF-kB signaling was tested in SFs, stimulated with 10 ng/ml TNF alpha (R&D Systems, # 210-TA) for 24 h.

### ReporterGene Assay

To measure NF-κB activity, SFs were transfected with 1.2 µg of pRL_GAPDH plus 1.8 µg of pGL4.32[*luc2P*/NF-κB-RE/Hygro] vector (Promega) or pGL4.27[luc2P/minP/Hygro] vector (Promega), using Nucleofector technology (Amaxa/Lonza). At 24 h after transfection, SFs were stimulated with EV in the presence or absence of 200 ng/mL TRAIL for 6 h, detached with trypsin and lysed in 1× Passive Lysis Buffer (Promega). Firefly luciferase activity was measured with a Dual Luciferase Reporter Assay System (Promega) and normalized to the activity of *Renilla* luciferase.

### Quantitative Real-time Polymerase Chain Reaction

Total RNA was isolated from SFs and U937 cells using miRNeasy Mini Kit (Qiagen) including on-column DNase I (Qiagen) digestion. 300 ng of total RNA was reverse transcribed using Random Hexamers and MultiScribe Reverse Transcriptase (Applied Biosystems/Thermo Fisher Scientific). The expression of proinflammatory genes (*IL-6, IL-8*) and antiviral genes [*MDA5*, retinoic acid-inducible gene 1 (*RIG-I*), *TLR3*, interferon beta (*IFNB*)] was measured by SYBR Green or TaqMan qPCR (7500 or 7900HT real-time PCR systems, Life Technologies) with normalization to *18S rRNA*, glyceraldehyde 3-phosphate dehydrogenase (*GAPDH)* or hypoxantine-guanine phosphoribosyltransferase (*HPRT1*) as indicated in respective figure legends. No template control samples, dissociation curves and samples containing the untranscribed RNA were measured in parallel. The primer sequences are given in Table S1 in Supplementary Material; except primers for *18S rRNA* and *IFNB* (Applied Biosystems). The expression of target mRNAs was determined using the comparative threshold cycle method as described in Ref. (27).

### Enzyme-Linked Immunosorbent Assay

The secretion of IL-6 and IL-8 into supernatants of SFs was measured with the human IL-6 and IL-8 ELISA sets (BD Biosciences), according to the manufacturer’s instructions.

### The Association of Poly(I:C) with U937 Cells and U937 Cell-Derived EV

To study the surface binding and internalization of Poly(I:C) into U937 cells and their cognate EV, 750,000 U937 cells were stimulated with 5 µg/ml Poly(I:C) HMW Fluorescein (InvivoGen, # tlrl-picf) or 5 µg/ml Poly(I:C) HMW Rhodamine (InvivoGen, # tlrl-picr) for 16 h. U937 cells and EV pellets were thoroughly washed and the presence of fluorescently labeled Poly(I:C) was measured by FACSCalibur flow cytometer (BD Biosciences). To assess whether Poly(I:C) can directly interact with EV, conditioned medium from unstimulated U937 cells was centrifuged to remove cells and cellular debris. Cell-free medium, containing Con EV was then incubated with 5 µg/ml Poly(I:C) HMW Rhodamine (InvivoGen, # tlrl-picr) for 120 min, 37°C, 5% CO_2_ followed by the isolation of EV and detection of Rhodamine Poly(I:C) by FACSCalibur flow cytometer (BD Biosciences).

To determine whether “vesicular” Poly(I:C) can be degraded by RNase, U937 cell-derived Fluorescein Poly(I:C) EV and Con EV, preincubated with Rhodamine Poly(I:C), were digested with RNase III (*E. coli*, Applied Biosystems/Ambion) for 2 h at 37°C according to the manufacturer’s protocol. Additionally, Poly(I:C), Rhodamine Poly(I:C) and Fluorescein Poly(I:C) were digested in tube with RNase III (*E. coli*, Applied Biosystems/Ambion) for 2 h at 37°C according to the manufacturer’s protocol. The efficiency of digestion of “vesicular” and “soluble” Poly(I:C) was assessed by flow cytometry (FACSCalibur, BD Biosciences) and agarose gel electrophoresis (using 1kB GeneRuler DNA ladder, Thermo Fisher Scientific). For flow cytometry analysis, 10 µg/ml of HMW Fluorescein Poly(I:C) was digested by 0.2 U/μl RNAse III.

### Transfer of Poly(I:C) to SFs *via* EV

To study the transfer of Poly(I:C) to SFs *via* EV, SFs were treated for 24 h with EV released from U937 cells upon stimulation with 5 µg/ml Poly(I:C) HMW Rhodamine or Poly(I:C) HMW Fluorescein. SFs were treated also with the Sup from the last washing of EV pellets or were stimulated with 5 µg/ml Rhodamine Poly(I:C) or 10 µg/ml Fluorescein Poly(I:C) for 24 h.

The presence of fluorescently-labeled Poly(I:C) in SFs was determined by flow cytometry and confocal microscopy. For flow cytometry, SFs were detached using the accutase, thoroughly washed with DPBS and immediately analyzed by FACSCalibur flow cytometer (BD Biosciences).

For confocal microscopy SFs, cultured in chamber slides (Lab-Tek; Nunc), were thoroughly washed with DPBS and fixed with 4% paraformaldehyde for 20 min RT. The slides were blocked with 1% bovine serum albumin/5% human serum for 40 min and incubated with FITC Mouse Anti-Human CD90 (BD Pharmingen, # 555595, 10 µg/ml, CD90 is a fibroblast surface marker) or FITC Mouse IgG1 κ isotype control antibodies (BD Pharmingen, # 555748, 10 µg/ml) for 1 h at RT. The nuclei were stained with DAPI (Sigma-Aldrich). Slides were covered with fluorescence mounting medium (Dako Cytomation). The images were taken by confocal laser scanning microscope Leica SP5 (Leica Microsystems) using the LAS AF software (Leica Microsystems).

### Flow Cytometry Analysis of U937 and PBMCs

U937 cells and PBMCs were analyzed on FACSCalibur flow cytometer (BD Biosciences). Fc receptors were preblocked with 10% FCS-containing DPBS for 30 min, 4°C. Cells were labeled with PE Mouse Anti-Human CD11b/Mac-1 (BD Pharmingen, #555388, 10 µg/ml), PE Mouse Anti-Human CD14 (BD Pharmingen, # 555398, 10 µg/ml) or PE Mouse IgG1 κ Isotype control (BD Pharmingen, #555749, 10 µg/ml) for 45 min, 4°C in the dark, thoroughly washed and analyzed.

### Western Blot

Cells were lysed in ice cold RIPA buffer and the insoluble material was removed by centrifugation at 12,000 *g*, 10 min. Whole cell lysates were separated on 10% SDS-polyacrylamide gels and electroblotted onto nitrocellulose membranes (Amersham Protran, GE Healthcare). Membranes were blocked in 5% (weight/volume) nonfat milk in TBS-T (20 mM Tris base, 137 mM sodium chloride, 0.1% Tween 20, pH 7.6) for 1 h. Western blots were performed using rabbit anti Caspase 3 antibodies (Cell Signaling, #9662, 1:1,000), which detect both cleaved and uncleaved forms of caspase 3, and mouse anti α-tubulin antibodies (Abcam, #ab7291, 1:10,000). Secondary antibodies conjugated with horseradish peroxidase were from Jackson ImmunoResearch (#111-036-047 and #115-036-062, 1:10,000). Protein bands were visualized using the enhanced chemiluminescence Western blot detection reagent (GE Healthcare) and the Fusion Fx Imager/Fusion software (Vilber Lourmat). Densitometry analysis of protein bands was carried out using the Bio-ID software (Vilber Lourmat). For quantification of Western blots, the levels of cleaved caspase 3 were normalized to the levels of uncleaved caspase 3.

### Annexin V Apoptosis Assay

U937 and SFs were washed with DPBS and 1 × 10^6^ cells/ml were resuspended in Annexin V binding buffer (BD Biosciences). Cells were labeled with FITC Annexin V (5 µl, BD Pharmingen, #556419) and propidium iodide (PI, Sigma-Aldrich) in the dark for 15 min, RT and analyzed by flow cytometry (FACSCalibur; BD Biosciences). Cells stained with Annexin V alone, PI alone and unstained cells were used to set up flow cytometry settings.

### Statistical Analysis

Data were analyzed with GraphPad Prism version 7.0. The distribution of data was tested with Kolmogorov–Smirnov and D’Agostino and Pearson omnibus normality tests for small and large samples, respectively. Multiple group comparisons were performed by one-way ANOVA with Tukey’s, Dunnett’s, or Sidak’s multiple comparisons tests (normal distribution) with or without Geisser–Greenberg correction for unequal sphericity or Friedmann test with Dunn’s multiple comparisons test (distribution not normal). Paired samples were compared with two-tailed paired *t*-test (normally distributed data) or Wilcoxon signed rank test (distribution not normal). *P*-value <0.05 was considered statistically significant. Heatmaps of experimental data (details provided in respective figure legends) were produced with Shiny by R Studio, developed by Functional Genomics Center Zurich, University of Zurich and ETH Zurich, Switzerland, using row scaling transformation of data.

## Results

### Characterization of U937 Cell-Derived EV

**Figure 1:**
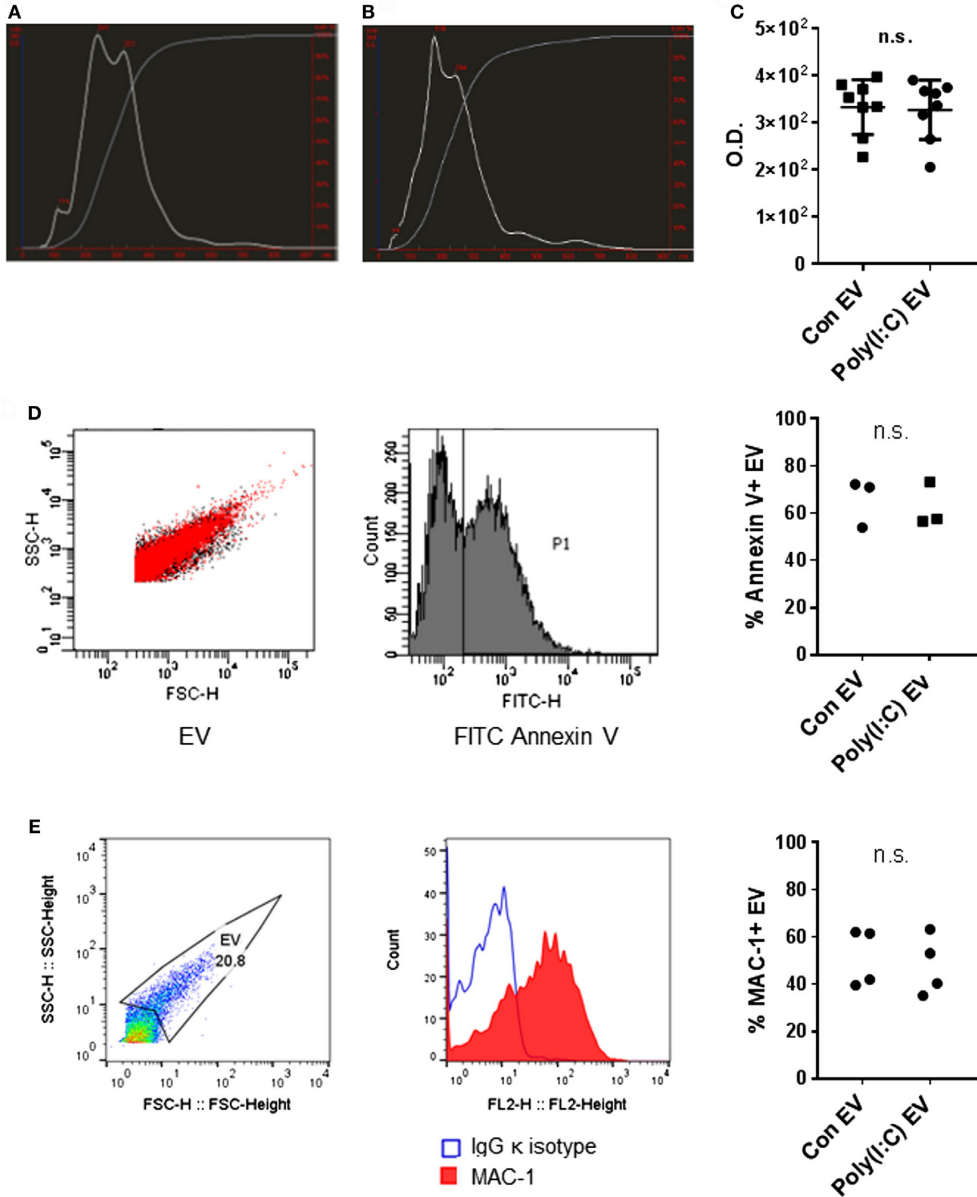
Characterization of U937 cell-derived extracellular vesicles (EV). Size distribution of particles (white line) and cumulative percentage of particles under given size (gray line) in EV preparations from **(A)** untreated U937 cells and **(B)** Poly(I:C)-stimulated U937 cells as detected by nanoparticle tracking analysis, shown is one of *n* = 2 biological replicates per condition. **(C)** Total protein amount in EV from untreated U937 cells (Con EV) and Poly(I:C)-stimulated U937 cells [Poly(I:C) EV] as measured by BCA Protein Assay, *n* = 8 biological replicates, mean ± SD. **(D)** Binding of FITC Annexin V to the surface of Con EV and Poly(I:C) EV with SSC-H/FSC-H profile of EV and histogram of FITC-labeled Annexin V binding, shown is one sample of *n* = 3 biological replicates per condition. Percentage of Annexin V-positive EV as measured by flow cytometry. **(E)** The expression of MAC-1 on the surface of Poly(I:C) EV with SSC-H/FSC-H profile of EV and FL2 histogram of MAC-1 positivity, as measured by flow cytometry. Shown is one sample of *n* = 4 biological replicates per condition. Percentage of MAC-1-positive EV as measured by flow cytometry. Statistics: **(C–E)** two-tailed paired *t*-test, ns, not significant.

We analyzed the size distribution and the amount of EV released from U937 cells using the NTA. NTA efficiently detects particles with diameters up to 1 µm, whereas Brownian motion of larger particles such as apoptotic bodies is slower and they are less readily detected (28). Our differential centrifugation protocol should predominantly isolate EV in the size range of microvesicles (0.1–1 μm) (29) while lacking exosomes (50–100 nm) that sediment at high centrifugation forces. Our protocol should also deplete apoptotic bodies (1–5 μm) (29) through a precentrifugation step at 1,600 *g* (30, 31). Accordingly, particles with sizes of microvesicles predominated in the EV preparations from unstimulated U937 cells (Con EV) [Figure 1A, median (10th, 90th percentile): 218 nm (114, 371 nm) and 227 (131, 368 nm), *n* = 2] and Poly(I:C)-stimulated U937 cells [Poly(I:C) EV] [Figure 1B, 346 nm (217, 504 nm) and 287 (187, 413 nm), *n* = 2]. A prominent peak of smaller particles was present in Con EV pellets (Figure 1A). In all, these results suggested that Con EV and Poly(I:C) EV might differ in composition or origin.

As measured by NTA, Poly(I:C) EV pellets contained smaller amounts of particles (5.20 × 10^9^/ml, 4.26 × 10^9^/ml, *n* = 2) compared with Con EV pellets (6.73 × 10^9^/ml, 5.98 × 10^9^/ml, *n* = 2). In contrast, the total protein content did not differ between Poly(I:C) EV and Con EV pellets (Figure 1C). Stimulation with Poly(I:C) increases the release of EV from different cell types as measured by flow cytometry (32, 33). Poly(I:C) enhances apoptosis in a variety of cell types (34, 35) including U937 cells (Figure S1A in Supplementary Material), thereby enriching cell supernatants for apoptotic vesicles, including apoptotic bodies. A depletion of apoptotic bodies by our protocol and their better visibility on flow cytometer could contribute to detecting the smaller amounts of particles in Poly(I:C) EV pellets by NTA compared with flow cytometry (32, 33). Alternatively, enhanced aggregation of particles in Poly(I:C) EV pellets might diminish the particle numbers and increase the particle size, while the total protein content would not change.

The International Society for Extracellular Vesicles set the guidelines on minimal experimental requirements for identifying EV, including the detection of phosphatidylserine and cell surface markers on vesicular membranes (36). As detected by flow cytometry, U937 cell-derived Con EV and Poly(I:C) EV bound similar amounts of Annexin V (Figure 1D) and expressed similar levels of the U937 cell surface molecule MAC-1 (Figure 1E), also known as integrin subunit alpha M (ITGAM) or CD11B, which is a cell surface receptor for extracellular dsRNA (37). Untreated and Poly(I:C)-treated U937 cells expressed similar levels of MAC-1 on their surface (Figure S1B in Supplementary Material).

Altogether, these measurements showed that Poly(I:C) EV and Con EV differ in size while exhibiting similar Annexin V binding and MAC-1 expression on the surface.

### Poly(I:C) EV Mimic the Proinflammatory and Antiviral Responses Induced by Poly(I:C)

**Figure 2:**
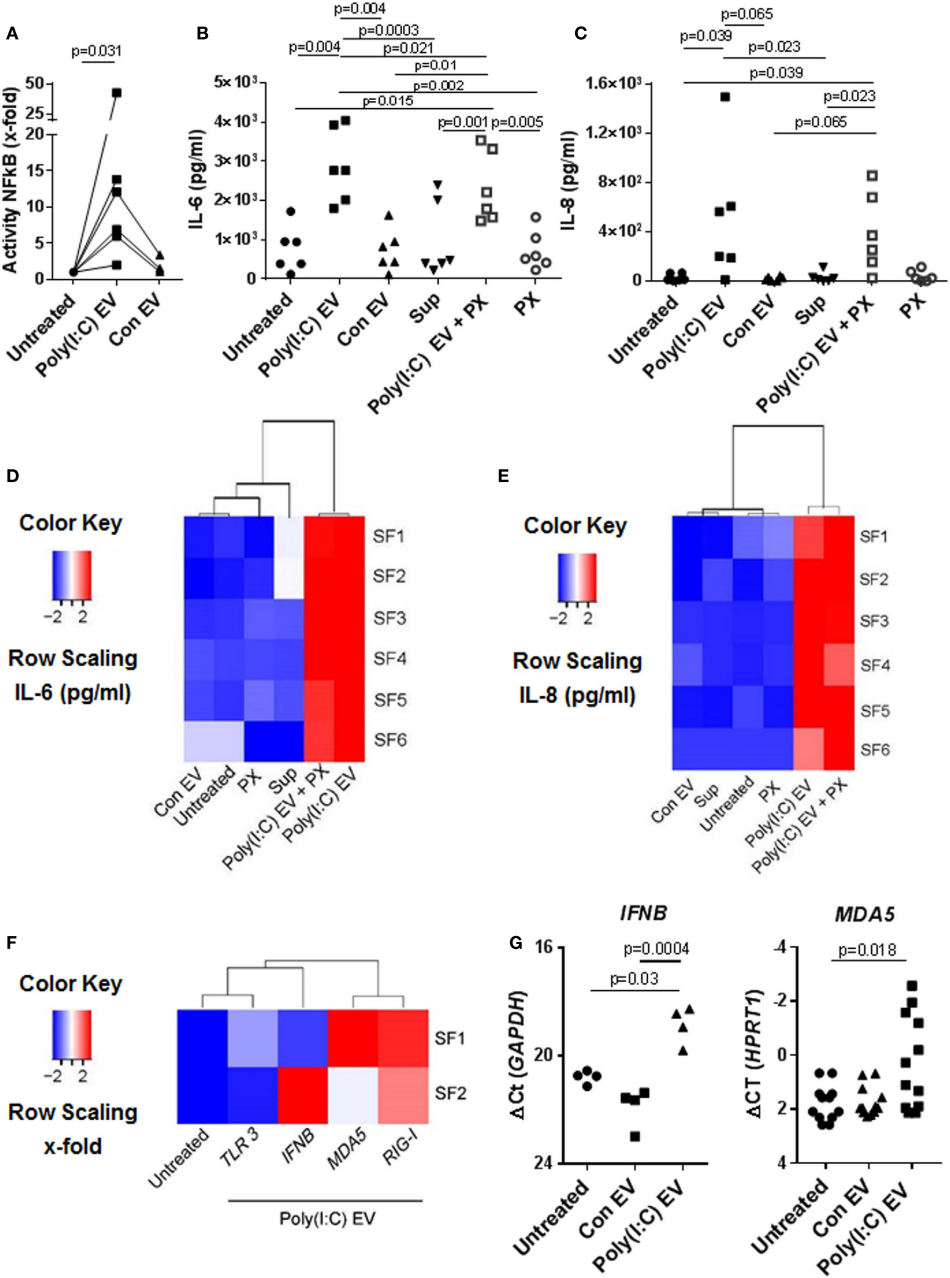
Extracellular vesicles (EV) derived from Poly(I:C)-stimulated U937 induce proinflammatory and antiviral responses in synovial fibroblasts (SFs). **(A)** The activity of NF-κB in synovial fibroblasts cocultured with EV derived from unstimulated U937 cells (Con EV) or Poly(I:C)-stimulated U937 cells [Poly(I:C) EV], as measured by a Dual Luciferase Reporter Assay System and expressed as x-fold induction compared to untreated cells, shown are biological replicates. Secretion of **(B)** IL-6 and **(C)** IL-8 proteins (pg/ml) into supernatants of synovial fibroblasts as measured by ELISA (biological replicates). Polymyxin B (PX) was used to control for LPS contamination. Supernatants from the last washing step of Poly(I:C) EV pellets (supernatants) were used to control for the carryover of soluble Poly(I:C) or U937-derived mediators. **(D,E)** Heatmaps show clustering of proinflammatory responses in synovial fibroblasts under different experimental conditions based on the IL-6 and IL-8 ELISA [see **(B,C)**]. **(F)** Heatmap demonstrates the clustering of antiviral gene responses in synovial fibroblasts cocultured with Poly(I:C) EV (qPCR, x-fold induction as compared with unstimulated cells, screening experiment with *n* = 2 biological replicates). **(G)** The expression of *MDA5* and *IFNB* in synovial fibroblasts cocultured with Con EV or Poly(I:C) EV, qPCR data expressed as ΔCt with normalization to *GAPDH* and *HPRT1*, respectively, shown are biological replicates. In the screening experiment, *MDA5* and *IFNB* showed variable induction upon coculture of synovial fibroblasts with Poly(I:C) EV and were thus measured in a larger cohort of synovial fibroblasts. Statistics: **(A)** Wilcoxon signed rank test, **(B)** one-way ANOVA with Tukey’s multiple comparison test and Geisser-Greenhouse correction for unequal sphericity, **(C)** Friedmann test with Dunn’s multiple comparisons test, **(G)** one-way ANOVA with Tukey’s multiple comparisons test (*IFNB*), Friedmann test with Dunn’s multiple comparisons test (*MDA5*).

Previous studies have shown that immune cell-derived EV, including EV from U937 cells, released *ex vivo* upon stimulation with proinflammatory mediators, enhance the matrix-destructive and proinflammatory properties of SFs (9). Besides, a transfer of cellular RNA *via* EV was shown sufficient for inducing the NF-kB signaling in recipient human embryonic kidney 293T cells (38). Here, we demonstrate that SFs, cocultured with U937 cell-derived Poly(I:C) EV activated signaling through the NF-kB pathway (Figure 2A), which coincided with the increased expression of proinflammatory genes (Figures S2A,B in Supplementary Material) and the enhanced secretion of IL-6 (Figure 2B; Figure S2A in Supplementary Material) and IL-8 (Figure 2C; Figure S2B in Supplementary Material) proteins into cell culture media. By using a set of negative controls in our experiments, including Sup controls (Figures 2B,C), FCS EV controls (Figure S2C in Supplementary Material), and Con EV (Figures 2B,C), we ruled out the carryover of soluble Poly(I:C) and U937-derived mediators *via* EV pellets, concluding thereby that the observed proinflammatory effects are specifically attributed to Poly(I:C) EV.

Polymyxin B strongly inhibited the LPS-induced secretion of IL-6 from SFs (Figure S2D in Supplementary Material), but had a limited effect on the IL-6 secretion induced with Poly(I:C) EV (Figure 2B). This argued against the presence of contaminating LPS in Poly(I:C) EV pellets and rather suggested a possible mild negative effect of Polymyxin B on the integrity of vesicular membrane (39). SFs cocultured with either Poly(I:C) EV or Poly(I:C) EV plus Polymyxin B clustered together based on the magnitude of their proinflammatory responses (Figures 2D,E), but clearly diverged from SFs treated with negative controls, which clustered together with untreated SFs (Figures 2D,E).

In addition to enhancing the proinflammatory responses, Poly(I:C) EV increased the expression of antiviral genes in SFs. The expression of genes such as *IFNB, MDA5*, and *RIG-I* was upregulated, while *TLR3* mRNA expression remained rather unchanged as shown by the clustering of transcriptional responses in SFs upon Poly(I:C) EV coculture (Figure 2F) as well as gene expression measurements in a larger set of SFs (Figure 2G).

Collectively, these results demonstrated that gene expression changes in the presence of Poly(I:C) EV were largely reminiscent of the transcriptional activation of SFs with Poly(I:C) (23, 40, 41). These effects of Poly(I:C) EV on SFs could reflect the transfer of Poly(I:C)-induced molecules from U937 cells to SFs *via* EV as shown for ovarian carcinoma (HEY) cell-derived Poly(I:C)-induced exosomes (15). Yet, we did not detect *IL-6* mRNA in U937 cells under our qPCR conditions and the levels of *IL-8, MDA5*, and *RIG-I* mRNAs in U937 cells did not alter upon Poly(I:C) stimulation (Figure S2E in Supplementary Material), indicating that both Con EV and Poly(IC) EV could shuttle these transcripts to SFs. The exclusive activation of SFs by Poly(I:C) EV thus suggested that Poly(I:C) EV might capture and transfer Poly(I:C) to SFs, thereby activating the dsRNA signaling pathways.

### U937 Cell-Derived EV Capture Poly(I:C)

**Figure 3:**
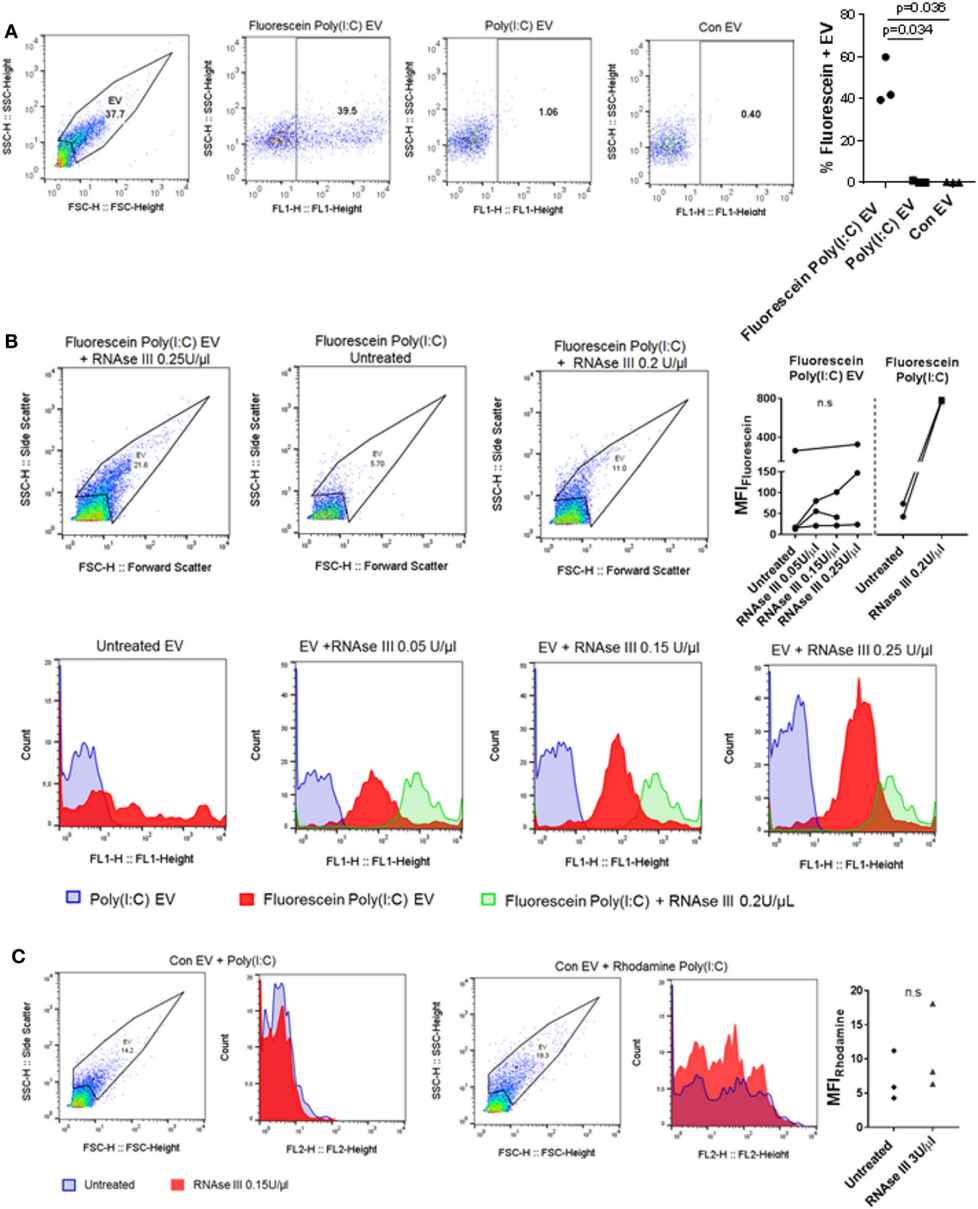
U937 cell-derived extracellular vesicles (EV) incorporate Poly(I:C) and may protect Poly(I:C) from degradation with RNase III. **(A)** The presence of Fluorescein Poly(I:C) in U937-derived EV. SSC-H/FSC-H and SSC-H/FL1 profiles of EV with percentage of gated events, as measured by flow cytometry. Shown are a representative sample of *n* = 3 biological replicates and quantification of flow cytometry data as percentage of Fluorescein Poly(I:C)-positive EV. Control EV (Con EV)-derived from untreated U937 cells. **(B)** Detection of soluble and vesicular Fluorescein Poly(I:C) digested or not with RNAse III. SSC-H/FSC-H profiles and FL1 histograms of EV and Poly(I:C), as measured by flow cytometry. Shown is one of *n* = 3–4 biological replicates for EV and one of *n* = 2 replicates for soluble Fluorescein Poly(I:C). Quantification of changes in median fluorescence intensities (MFI) of vesicular and soluble Fluorescein Poly(I:C) in the presence and absence of RNAse III. **(C)** Association of control EV (Con EV) with Poly(I:C) or Rhodamine Poly(I:C) in the presence or absence of RNase III, as measured by flow cytometry, shown is one from *n* = 3 biological replicates. Quantification of MFI changes in Rhodamine Poly(I:C) EV in the presence of RNAse III. Statistics: **(A,B)** one-way ANOVA with Tukey’s multiple comparisons test, **(C)** two-tailed paired *t*-test, ns, not significant.

Extracellular nucleic acids, such as dsDNA, readily associate with EV (42), and EV contain a diversity of small and long intracellular RNA species (with lengths up to 4,000 bases) (30). Vesicular RNA can be transferred to recipient cells in cocultures and tissue microenvironments altering thereby recipient cell functions (38, 43, 44). Based on these studies, we speculated that EV could capture at least some of the HMW Poly(I:C) molecules (1.5–8 kb) and transfer them to SFs. To study the capturing of Poly(I:C) in vesicles, we stimulated U937 cells with Rhodamine Poly(I:C) or Fluorescein Poly(I:C); we digested Poly(I:C) EV with RNAse III in a subset of experiments. We showed that fluorescently labeled Poly(I:C) was present in U937 cells (Figure S3A in Supplementary Material) and their cognate EV (Figure 3A; Figures S3B,C in Supplementary Material), and variable proportions of EV, as detected by flow cytometry, contained Poly(I:C) (Figure 3A; Figures S3B,C in Supplementary Material). This finding is consistent with recent studies showing the capture of Poly(I:C) as well as other TLR ligands in exosomes from dendritic cells that were exposed to Poly(I:C) or other TLR ligands (45, 46). We also analyzed soluble HMW Fluorescein Poly(I:C) by flow cytometry. This analysis demonstrated that vesicular and soluble fluorescent Poly(I:C) have comparable median florescence intensities, but distinct forward/side scatter characteristics, which enables their discrimination by flow cytometry (Figure 3B). Specifically, soluble Fluorescein Poly(I:C) was detected largely within cytometer noise and only few fluorescent events spilled into the EV flow cytometry window.

Extracellular RNA, present in EV, bound to cell surface molecules, or complexed with Argonaut proteins, can be protected from RNAse degradation (47–49). To investigate RNAse sensitivity of the EV-contained Poly(I:C) we used flow cytometry and showed that EV-contained Poly(I:C) is at least partially resistant to degradation with RNAse III (Figure 3B). In contrast, this nuclease could efficiently degrade soluble fluorescent Poly(I:C) (Figures S3D,E in Supplementary Material). RNAse-digested soluble Fluorescein Poly(I:C) spilled to a greater extent into the EV flow cytometry window compared with nondigested soluble Fluorescein Poly(I:C) and was characterized with a large (more than 15-fold) increase in median fluorescence intensity (MFI) compared to nondigested soluble Fluorescein Poly(I:C) (Figure 3B). In contrast, MFIs of vesicular Fluorescein Poly(I:C) increased only moderately and non-significantly in the presence of increasing concentrations of RNase III (Figure 3B). This indicated that RNAse III-treated Fluorescein Poly(I:C) EV contained a limited amount of degraded Fluorescein Poly(I:C) and that EV at least partially protected Poly(I:C) from degradation with RNAse III. Additionally, we removed cells and cell debris from the cell culture media of unstimulated U937 cells *via* centrifugation and incubated the resulting cell-free media with fluorescently-labeled Poly(I:C). These experiments showed that Poly(I:C) readily associated with Con EV in the cell-free media from unstimulated U937 cells (Figure 3C). MFI of vesicular Rhodamine Poly(I:C) did not increase significantly in the presence of RNAse III (Figure 3C). This further demonstrated that in a complex with EV, Poly(I:C) was rather protected from RNAse III degradation (Figure 3C).

Collectively, these results demonstrated that Poly(I:C) can associate with EV, either trapped intravesicularly or bound to the vesicular surface in a manner that makes it rather resistant to RNase III degradation.

How cells sense and internalize extracellular dsRNA to signal through the intracellularly located dsRNA sensors is not completely understood (50). The cell surface receptor CD11b/CD18 (Mac-1) is one of the sensors for extracellular Poly(I:C) and participates in Poly(I:C) internalization into mouse macrophages (37). We showed that MAC-1 was present on the surface of U937 cells (Figure S1B in Supplementary Material) and U937-cell derived EV (Figure 1E). Similar amounts of MAC-1 were detected in unstimulated and Poly(I:C)-stimulated U937 cells (Figure S1B in Supplementary Material) as well as their cognate EV (Figure 1E). The presence of MAC-1 on U937 cells and EV suggested that MAC-1 may be one of the receptors contributing to the surface binding as well as trapping of Poly(I:C) within U937 cells and their EV.

### Poly(I:C) EV Transfer Poly(I:C) to SFs

**Figure 4:**
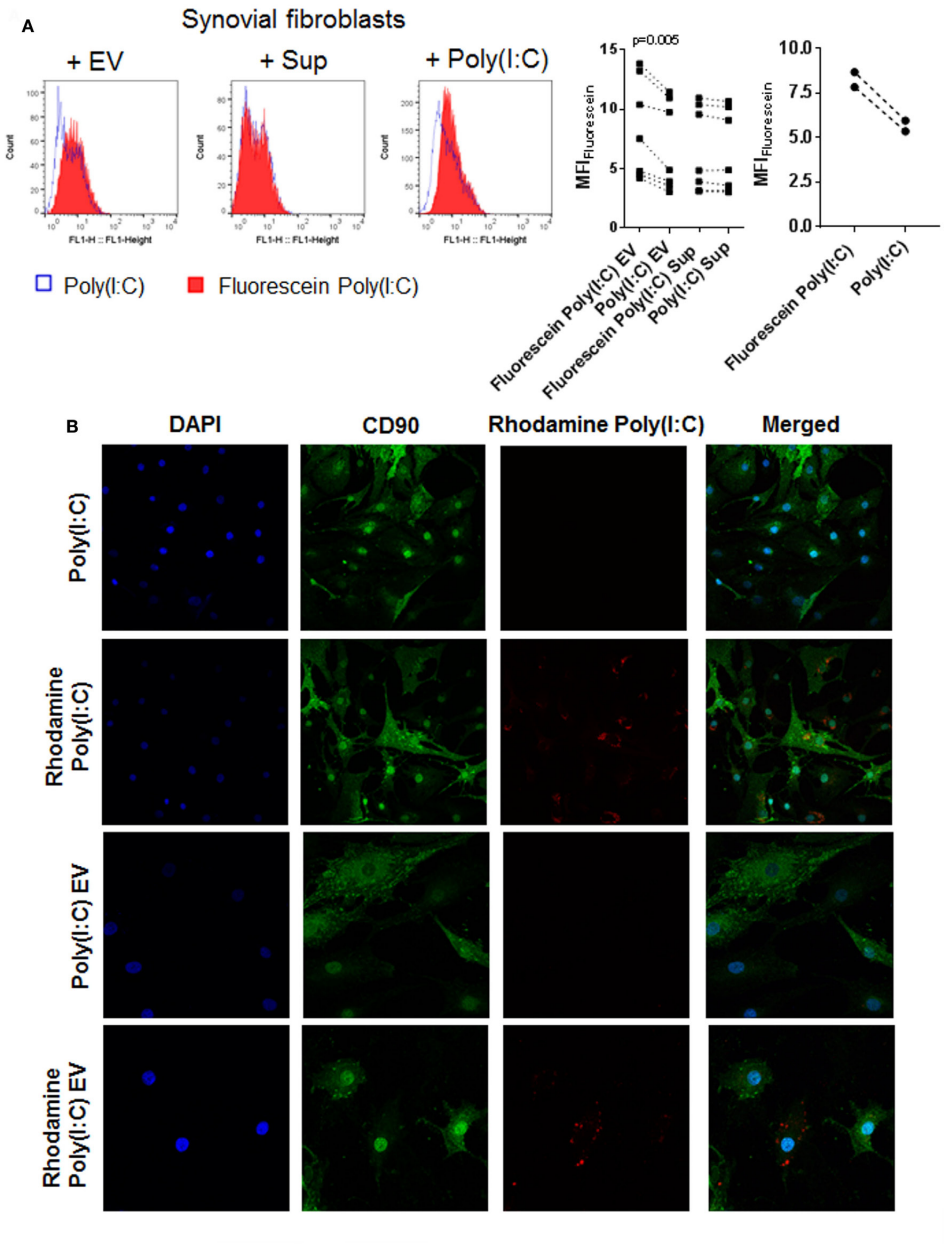
U937 cell derived extracellular vesicles (EV) shuttle Poly(I:C) to synovial fibroblasts. **(A)** The presence of Fluorescein Poly(I:C) in synovial fibroblasts either cocultured with U937 cell-derived Fluorescein Poly(I:C) EV or Poly(I:C) EV, or treated with supernatants from the last washing step of the respective EV pellets or stimulated with Poly(I:C)/Fluorescein Poly(I:C). Sup were used to control for the contamination with soluble Poly(I:C). Shown are FL1 histogram of fluorescein fluorescence in a representative sample and quantification of changes in median fluorescence intensities (MFI) of synovial fibroblasts upon different treatments. **(B)** Confocal microscopy on synovial fibroblasts treated with Poly(I:C) or Rhodamine Poly(I:C) or cocultured with U937 cell-derived Poly(I:C) EV or Rhodamine Poly(I:C) EV for 24 h. Nuclei-DAPI (blue), FITC Mouse Anti-Human CD90 (green, fibroblast marker), Rhodamine Poly(I:C) (red). Magnification: 20× [Poly(I:C), Rhodamine Poly(I:C)] and 40× [Poly(I:C) EV, Rhodamine Poly(I:C) EV]. Statistics: **(A)** one way ANOVA with Sidak’s multiple comparison test and Geisser-Greenhouse correction for unequal sphericity.

Extracellular vesicles can transfer RNA between cells and thereby influence the recipient cell’s functions (43, 51). To explore whether EV can shuttle Poly(I:C) to SFs, we cocultured SFs with EV isolated from U937 that were stimulated with fluorescently labeled Poly(I:C). Additionally, SFs were directly treated with fluorescently-labeled Poly(I:C). As demonstrated by flow cytometry (Figure 4A) and fluorescence confocal microscopy (Figure 4B; Figure S4A in Supplementary Material), Poly(I:C) EV transferred Poly(I:C) to SFs. The distribution of Rhodamine Poly(I:C) differed in SFs stimulated with Poly(I:C) as compared with Poly(I:C) EV (Figure 4B; Figure S4A in Supplementary Material). The precise 3D localization (intra/extracellular) of vesicular Poly(I:C) was difficult to determine because of the flat fibroblast morphology (Figure S4A in Supplementary Material). Nevertheless, the recapitulation of Poly(I:C) responses by Poly(I:C) EV (Figure 2; Figure S2 in Supplementary Material) clearly showed that vesicular Poly(I:C) can efficiently induce Poly(I:C) responses in SFs. In these experiments, fluorescently labeled Poly(I:C) was not detected in SFs treated with Sup from the last washing step of Poly(I:C) EV (Figure 4A), thereby excluding potential carryover of soluble Poly(I:C) *via* EV pellets. Additionally, Fluorescein Poly(I:C) EV were readily detectable in the Sup of SFs at 24 h after starting the cocultures (Figure S4B in Supplementary Material), demonstrating the stability of vesicular Poly(I:C) in the extracellular space over time.

In summary, these results suggested that, in microenvironments that contain extracellular dsRNA (e.g., endogenous dsRNA released from damaged tissues), monocyte-derived EV could play a prominent role in the intercellular shuttling of dsRNA and cellular responses to extracellular dsRNA.

### Poly(I:C) EV Deliver a Prosurvival Signal during Death Receptor Induced-Apoptosis

**Figure 5:**
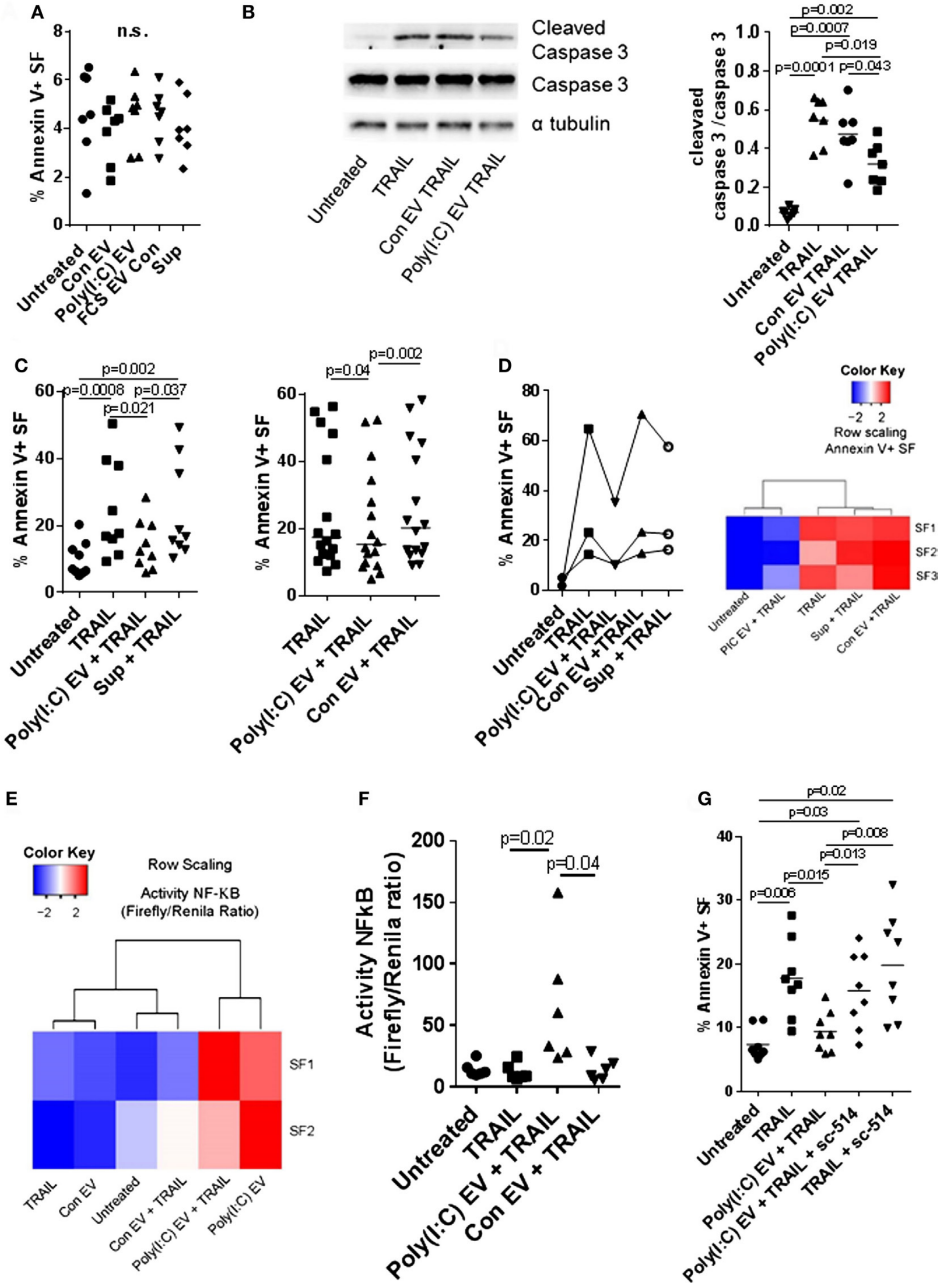
Extracellular vesicles (EV) derived from Poly(I:C)-stimulated U937 cells have antiapoptotic effects in synovial fibroblasts. **(A)** Percentage of apoptotic Annexin V-positive synovial fibroblasts upon treatment with U937 cell-derived EV, as measured by flow cytometry (representative flow cytometry charts are provided as Figure S5C in Supplementary Material). Supernatants from the last washing step of Poly(I:C) EV pellets and fetal calf serum EV control (FCS EV) were used to control for Poly(I:C) carryover and the effects of potential residual FCS-derived EV, respectively. **(B)** The cleavage of caspase 3 in synovial fibroblasts treated for 24 h with TRAIL ± U937 cell-derived EV. Shown is Western blot for caspase 3 and cleaved caspase 3 from one of *n* = 7 biological replicates with α tubulin as loading control. The nitrocellulose membrane from the same gel was used for detection of caspase3, cleaved caspase 3 and α tubulin with different exposure times for detection of each protein. Provided is merged image and the original gel images are provided as Figure S9 in Supplementary Material. Densitometry analysis of the cleaved caspase 3 bands was normalized to uncleaved caspase 3 bands. **(C)** Percentage of apoptotic Annexin V-positive synovial fibroblasts upon treatment with TRAIL ± U937 cell-derived EV or supernatants from the last washing step of Poly(I:C) EV pellets (supernatants), as measured by flow cytometry (representative flow cytometry charts are provided as Figure S6A in Supplementary Material). Shown are two different experimental setups with *n* = 9 and *n* = 16 biological replicates. **(D)** Percentage of apoptotic Annexin V-positive synovial fibroblasts, as measured by flow cytometry, upon treatment with TRAIL ± peripheral blood mononuclear cell (PBMC)-derived EV or supernatants from the last washing step of Poly(I:C) EV pellets (supernatants). Representative flow cytometry charts are provided as Figure S7A in Supplementary Material. PBMCs from healthy donors were cultured *ex vivo* in the presence or absence of Poly(I:C) for 16 h. Heatmap (row scaling) shows the clustering of TRAIL-induced apoptotic responses of synovial fibroblasts (SFs) under different experimental conditions based on the flow cytometry measurements of Annexin V binding. **(E,F)** The activity of NF-κB in synovial fibroblasts cocultured with U937 cell-derived EV ± TRAIL for 6 h, as measured by a Dual Luciferase Reporter Assay System. Data are expressed as the ratio of the activity of Firefly to Renilla luciferase in cells transfected with pRL_GAPDH plus pGL4.32[*luc2P*/NF-κB-RE/Hygro] or pGL4.27[luc2P/minP/Hygro] vectors for 30 h. Shown are two different experimental setups: **(E)** a screening experiment with *n* = 2 biological replicates and **(F)** a confirmatory experiment with *n* = 6 biological replicates. **(G)** Percentage of apoptotic Annexin V-positive synovial fibroblasts, as measured by flow cytometry, upon treatment with TRAIL ± Poly(I:C) EV and/or sc-514 (50 µM), the inhibitor of IKK-2, shown are biological replicates. Representative flow cytometry charts are provided as Figure S8A in Supplementary Material. Statistics: **(A)** one-way ANOVA with Dunnett’s multiple comparisons test and Geisser–Greenberg correction for unequal variances **(B,C,F)** Friedmann ANOVA with Dunn’s multiple comparisons test **(G)** one-way ANOVA with Tukey’s multiple comparisons test and Geisser–Greenberg correction for unequal variances. ns, not significant.

Poly(I:C) increases apoptosis of a variety of cell types (34, 35) including SFs (Figure S5A in Supplementary Material, in doses > 5 μg/ml). Intracellular delivery of very small amounts of Poly(I:C) by transfection with Lipofectamine 2000 was highly toxic for SFs (Figure S5B in Supplementary Material). In contrast, Poly(I:C) EV did not activate proapoptotic pathways in SFs (Figure 5A; Figure S5C in Supplementary Material). As estimated from the magnitude of *MDA5* and *IL-6* transcriptional responses of SF to direct stimulation with Poly(I:C) (Figures S5D,E in Supplementary Material), Poly(I:C) EV most likely transferred only a small amount of Poly(I:C) to SFs. Although these amounts were sufficient to induce the proinflammatory and antiviral responses, they might be too small to convey the proapoptotic effects of Poly(I:C).

Decreased cell death of SFs from RA joints in response to proapoptotic stimuli such as TRAIL could contribute to synovial hyperplasia and the formation of the invasive synovial tissue that destroys articular cartilage (52). To further explore the effects of Poly(I:C) EV on the apoptotic pathways in SFs, we treated SFs with the death receptor ligands TRAIL and FasL. Poly(I:C) EV decreased the TRAIL-induced apoptosis (Figures 5B,C; Figure S6A in Supplementary Material) and FasL-induced apoptosis of SFs (Figure S6B in Supplementary Material) as detected by Annexin V/PI apoptosis assay and Western blot measurement of cleaved caspase 3. Additionally, we performed a set of negative control experiments with Con EV and control Sup (Figures 5B,C; Figure S6A in Supplementary Material) as well as positive control experiments with Poly(I:C) transfection (Figure S5B in Supplementary Material), direct stimulation with Poly(I:C) (Figure S6C in Supplementary Material) or TNF EV (Figure S6D in Supplementary Material). Using these sets of experiments, we could exclude the possibility of antiapoptotic actions of contaminating soluble Poly(I:C) or U937-derived mediators and proposed that the observed prosurvival effects on death receptor-induced apoptosis are a specific action of Poly(I:C) EV. Therefore, the amount as well as the route of Poly(I:C) delivery to a cell (as part of a complex EV cargo) could be crucial in regulating the balance between prosurvival and proapoptotic actions of Poly(I:C).

All in all, these results showed that Poly(I:C) EV mimic the proinflammatory and antiviral responses of SFs induced by direct stimulation with Poly(I:C), yet, have unique prosurvival effects on death receptor-induced apoptosis which is in contrast to the high toxicity of transfected Poly(I:C).

### Poly(I:C) EV from PBMCs Reproduce the Prosurvival Actions of U937-Cell Derived Poly(I:C) EV

The interactions between monocytes or monocyte-derived EV and SFs can modulate synovial disease pathways in inflammatory arthritis (9, 52). U937 cells stimulated with TLR ligands are a relevant model for studying the effects of monocyte-derived EV in SFs (9); nevertheless, the analysis of these effects in primary human cells is desirable. To investigate the properties of EV from primary cells, we characterized the activity of EV from PBMCs cultured *ex vivo* in the presence or absence of Poly(I:C). We showed that PBMC-derived Poly(I:C) EV, but not Con EV or Sup controls, efficiently decreased TRAIL-induced apoptosis in SFs (Figure 5D; Figure S7A in Supplementary Material), displaying the antiapoptotic actions of U937 cell-derived Poly(I:C) EV. This finding showed that Poly(I:C) EV, derived from myeloid and/or lymphoid cells induced similar antiapoptotic responses in SFs, suggesting a general mechanism of EV, derived from these cells, in modulation of responses to dsRNA. Within the PBMC population, monocytes expressed high amounts of MAC-1, whereas lymphocytes expressed medium amounts of MAC-1 on their surface (Figure S7B in Supplementary Material). Accordingly, the MAC-1 high peak was not present in monocyte-depleted PBMCs (Figure S7B in Supplementary Material). This suggested that both monocyte- and lymphocyte-derived EV in the EV pellets from PBMCs could trap or interact with Poly(I:C).

### Activation of the NF-kB Pathway Confers Prosurvival Actions to Poly(I:C) EV

Inhibition of NF-kB signaling can sensitize a variety of cell types to apoptosis induced by TRAIL (53, 54). Here we showed that Poly(I:C) EV efficiently activated NF-kB signaling in SFs in the absence or presence of TRAIL (Figures 2A and 5E,F). Accordingly, SFs cocultured with Poly(I:C) EV ± TRAIL clustered together based on the magnitude of NF-kB signaling (Figure 5E), while diverging from SFs treated with Con EV and/or TRAIL that clustered with untreated cells (Figure 5E). Next, we inhibited the NF-kB activity with sc-514, an inhibitor of the inhibitor of nuclear factor kappa B kinase subunit beta (IKBKB, also known as IKK-2). Pretreatment of SFs with sc-514 impaired the prosurvival effects of Poly(I:C) EV during TRAIL-induced apoptosis (Figure 5G; Figure S8A in Supplementary Material), thus substantiating the role for NF-kB activation in the prosurvival actions of Poly(I:C) EV. A strong repression of the TNF-driven production of IL-6 in SFs (Figure S8B in Supplementary Material) confirmed the efficiency of sc-514 in repressing NF-kB signaling in SF.

In contrast to Poly(I:C) EV, transfected Poly(I:C) (Figure S5B in Supplementary Material) or direct stimulation with Poly(I:C) (Figure S6C in Supplementary Material) had no effect on TRAIL-induced apoptosis of SFs. Transfected Poly(I:C) was highly toxic with more than 90% cell death in SFs (Figure S5B in Supplementary Material). Meanwhile, direct stimulation of SFs with Poly(I:C) can activate the NF-kB signaling (23), however, it also enhanced apoptosis of SFs (Figure S5A in Supplementary Material). Increased apoptosis of SFs in the presence of high dose Poly(I:C) could mask the NF-kB-dependent reduction in TRAIL-induced apoptosis. In turn, small doses of Poly(I:C) might not be sufficient to activate SFs (Figures S5D,E in Supplementary Material) unless delivered to cells *via* EV (Figures 2B,C; Figures S5D,E in Supplementary Material).

Collectively, this indicates that Poly(I:C) EV efficiently deliver small amounts of Poly(I:C), which can selectively activate antiviral and proinflammatory responses in SFs, the latter contributing to the prosurvival effects of Poly(I:C) EV during TRAIL-induced apoptosis.

## Discussion

Extracellular vesicles are increasingly recognized as key players in the immune responses to TLR activation (11, 15, 16, 45, 46). Here we show that EV derived from monocyte U937 cells efficiently shuttle the TLR3 ligand Poly(I:C) to SFs and activate Poly(I:C)-induced signaling with enhanced proinflammatory and antiviral gene responses. Our experimental approach highlights the key importance of experimental controls that track the potential transfer of a stimulus [Poly(I:C)] *via* EV when studying the effects of stimulus-induced EV. We demonstrate that Poly(I:C) is present in EV released from Poly(I:C)-stimulated U937 cells but can also directly associate with EV in cell culture media from unstimulated U937 cells. These observations suggest that, in microenvironments (e.g., RA synovial joints) that are rich in extracellular RNA (20, 22) and monocyte-derived EV (55), extracellular RNA may directly interact with EV to become a bound constituent. Additionally, EV might shuttle the extracellular dsRNA from the sites of generation to distant sites, thereby inducing proinflammatory and antiviral responses in recipient cells at remote locations.

The capacity of extracellular dsRNA to transcriptionally activate target genes relies on its extracellular stability and efficient intracellular delivery. Our experiments demonstrate that, similar to RNA in circulating EV, Poly(I:C) in vesicle form is stable over time and is rather protected from RNAse III degradation; this stability contrasts with the susceptibility of free Poly(I:C) to enzyme degradation. This finding suggests that the association of dsRNA with monocyte-derived EV within the proinflammatory milieu of RA joints could potentially protect extracellular dsRNA from RNase degradation. Indeed, large amounts of extracellular RNA are present in the RA synovium despite increased activity of RNases in synovial fluid from RA joints (20, 56). In turn, OA joints exhibit increased RNAse activity but small amounts of extracellular RNA (20).

The entry of Poly(I:C) into cells and the responses of cells to Poly(I:C) can vary with a cell type and the molecular weight of Poly(I:C) (57). A number of cell surface receptors such as MAC-1 and class A scavenger receptors can participate in the extracellular recognition and/or cellular uptake of Poly(I:C) (37, 50), clathrin-dependent endocytosis (58), and raftlin (17, 57) can also have essential roles in cellular internalization of Poly(I:C). We and others (46) provide evidence that EV can play an important role in the cellular entry and intercellular transfer of dsRNA. This ability of EV for transferring RNA has important implications for understanding the biology of extracellular dsRNA and developing new therapy, for example in the design of exosome-based antitumor vaccines (45, 46, 59). dsRNA and EV seem to utilize similar mechanisms of cellular uptake. Clathrin and MAC-1 are present on neutrophil-derived EV and are involved in the internalization of neutrophil-derived EV into platelets (60). Blocking the clathrin- or MAC-1-dependent uptake of neutrophil-derived EV by using chlorpromazine and MAC-1 antibodies, respectively, significantly decreases the production of TxA_2_ in platelets (60). We demonstrated that, similar to neutrophil-derived EV, U937 cell-derived EV express MAC-1 on their surface, suggesting that MAC-1 might participate in EV loading and intercellular shuttling of Poly(I:C).

We showed that the proinflammatory and antiviral responses of SFs in cocultures with Poly(I:C) EV largely recapitulate the transcriptional activation of SFs directly stimulated with Poly(I:C) although *TLR3* mRNA expression does not increase. The induction of *MDA5* and *RIG-I* mRNAs upon direct stimulation of SFs with Poly(I:C) is much stronger compared with the induction of *TLR3* mRNA (41). This reduced response might explain the lack of transcriptional activation of the *TLR3* gene in SFs cocultured with Poly(I:C) EV, which shuttle rather a limited amount of Poly(I:C). In addition, EV are expected to transfer the U937-derived molecular cargo to SFs, including DNA, RNA, protein and lipids as shown for a variety of other target cell–EV interactions (10, 61, 62). U937 cell-derived molecules might directly configure fibroblast responses and might also fine-tune the actions of EV-delivered Poly(I:C), for example, by shuttling the myeloid cell-derived miR-223(63), which is predicted to target *TLR3* mRNA (64). EV released from hepatitis B virus-infected HepG2 hepatocyte cells transfer virus-induced microRNA, which inhibit the induction of IL12p40 upon stimulation of THP-1 macrophages with Poly(I:C) and CL097 (65). Additionally, Poly(I:C) EV might shuttle a distinct molecular cargo compared with Con EV, which might contribute to or independently activate SFs.

On the basis of our experiments, we argue that the antiviral and proinflammatory effects of Poly(I:C) EV which we describe here are primarily caused by the transcriptional activation of SFs with EV-delivered Poly(I:C). Autologous exosomes released from ovarian carcinoma (HEY) cells upon Poly(I:C) stimulation transfer the Poly(I:C)-induced transcripts to Poly(I:C) naive HEY cells, thereby recapitulating Poly(I:C)-driven cell activation (15). In contrast, we used U937 cells, a myeloid cell type, for EV production, but studied the effects of EV in SFs, a non-myeloid cell type. Myeloid and non-myeloid cells distinctly respond to Poly(I:C) of different molecular weights (57). Whereas RAW264.7 cells, THP-1 cells and human PBMCs are preferentially activated by low-molecular-weight (LMW) Poly(I:C) (~1–1.5 kb), HMW Poly(I:C) (>5 kb) elicits strong antiviral and cytokine responses in fibroblasts (57). Whether EV inherit cell-type specific preference for the size of Poly(I:C) molecules and can thereby invoke distinct responses in myeloid and non-myeloid cells remains to be uncovered. Our experiments show that, while HMW Poly(I:C) increases the apoptosis of U937 cells and SFs, only SFs upregulate the expression of antiviral and cytokine genes. This finding strongly suggests that the transfer of vesicular Poly(I:C) is the main factor in recapitulating Poly(I:C) responses in SFs cocultured with Poly(I:C) EV. Similarly, dendritic cell-derived exosomes can cross-present TLR ligands, thereby activating the bystander dendritic cells (46). Furthermore, EV from virus-infected cells shuttle viral RNA and elicit antiviral responses in recipient dendritic cells (66).

Extracellular vesicles share the extracellular space with soluble mediators and can modulate the effects of soluble molecules in proinflammatory microenvironments. EV derived from the acute lymphoblastic leukemia (CCRF) cells modify the actions of the proinflammatory cytokine TNF in U937 cells, resulting in antagonistic, additive or synergistic effects (13). Here we demonstrate that U937 cell-derived Poly(I:C) EV exhibit unique antiapoptotic actions in SFs during death receptor-induced apoptosis; this action depends on the Poly(I:C) EV-driven activation of NF-kB. These effects cannot be induced by Con EV, TNF EV or Poly(I:C) alone, but can be reproduced by PBMC-derived Poly(I:C) EV. This suggests that the presence of Poly(I:C) in the complex with EV rather than a specificity of a cell type determines the antiapoptotic actions of Poly(I:C) EV. Thus, in addition to altering the cellular responses to cytokines (13), EV modify also the actions of extracellular dsRNA. Poly(I:C) EV have a unique capacity to simultaneously promote the proinflammatory and antiapoptotic responses in SFs. The antiapoptotic effects sharply contrast the high toxicity of transfected Poly(I:C) and might be attributed to the route of delivery of Poly(I:C) (*via* EV), the complexing of Poly(I:C) with EV or the fine-tuning of Poly(I:C) actions by molecular cargo from U937 cells.

By their ability to transfer pathogen-associated molecular patterns (PAMP)-induced or DAMP-induced transcripts as well as PAMPs or DAMPs themselves, EV have properties consistent with an important role as messengers of an ongoing infection or tissue damage, communicating a danger signal to the host. Our work shows that, by shuttling dsRNA *via* EV, the cells of origin can reduce the need for an active production of danger signaling molecules such as proinflammatory cytokines and antiviral molecules. EV containing dsRNA can reproduce the antiviral and proinflammatory actions of dsRNA, nevertheless, these structures allow dsRNA to exert antiapoptotic effects on SFs. This unexpected inversion in the nature of dsRNA actions by EV might potentiate the pathogenicity of dsRNA *in vivo* by enhancing the expansion of the invasive synovial tissue in RA. As such, blocking the EV-dsRNA interactions could have therapeutic effects in arthritis. Future studies will define the contribution of EV to RA pathogenesis and effects of EV containing RNA in terms of proinflammatory and antiapoptotic actions.

## Ethics Statement

This study was approved by the local ethic committee of the University Hospital Zurich, Switzerland and was carried out in accordance with the recommendations of the local ethic committee of the University Hospital Zurich, Switzerland. All subjects gave written informed consent in accordance with the Declaration of Helsinki.

## Author Contributions

MF-B designed and performed the experiments, acquired and analyzed the data, and wrote the manuscript. DP participated in critical discussion of the data and drafting the manuscript. CK communicated with patients, obtained informed consents and synovial tissues, and drafted the manuscript. BM and RG were involved in the coordination of the study and drafting the manuscript. AJ set the basis of the project and participated in discussions. SG participated in project discussion, coordinated the project and drafted the manuscript. All authors have seen and approved the manuscript and its contents and are aware of the responsibilities connected to authorship.

## Conflict of Interest Statement

The authors declare that the research was conducted in the absence of any commercial or financial relationships that could be construed as a potential conflict of interest.

## Supplementary Material

The Supplementary Material for this article can be found online http://www.frontiersin.org/articles/10.3389/fimmu.2018.00028/full#supplementary-material.
