# Supplementary material for: TLR3 Ligand Poly(I:C) Exerts Distinct Actions in Synovial Fibroblasts When Delivered by Extracellular Vesicles
Source: Front Immunol. 2018 Jan 29;9:28. doi: 10.3389/fimmu.2018.00028 (PMC5797482; doi:10.3389/fimmu.2018.00028)
Supplement: Supplementary file 1 [file Presentation_1.PDF]

Supplementary Material

TLR3 ligand Poly(I:C) exerts distinct actions in synovial fibroblasts when delivered by extracellular vesicles

Mojca Frank-Bertoncelj[1,*] David S Pisetsky[2,3], Christoph Kolling[4], Beat A Michel[1], Renate E Gay[1], Astrid Jungel[1], Steffen Gay[1]

[1]Center of Experimental Rheumatology, Department of Rheumatology, University Hospital Zurich, Schlieren, Switzerland

[2] Duke University Medical Center, Department of Medicine, Durham, NC, USA

[3] Medical Research Service, Durham Veterans Administration Medical Center, Durham, NC, USA

[4] Schulthess Clinic, Zurich, Switzerland, Country

*** Correspondence:**
Mojca Frank-Bertoncelj
mojca.frank@usz.ch

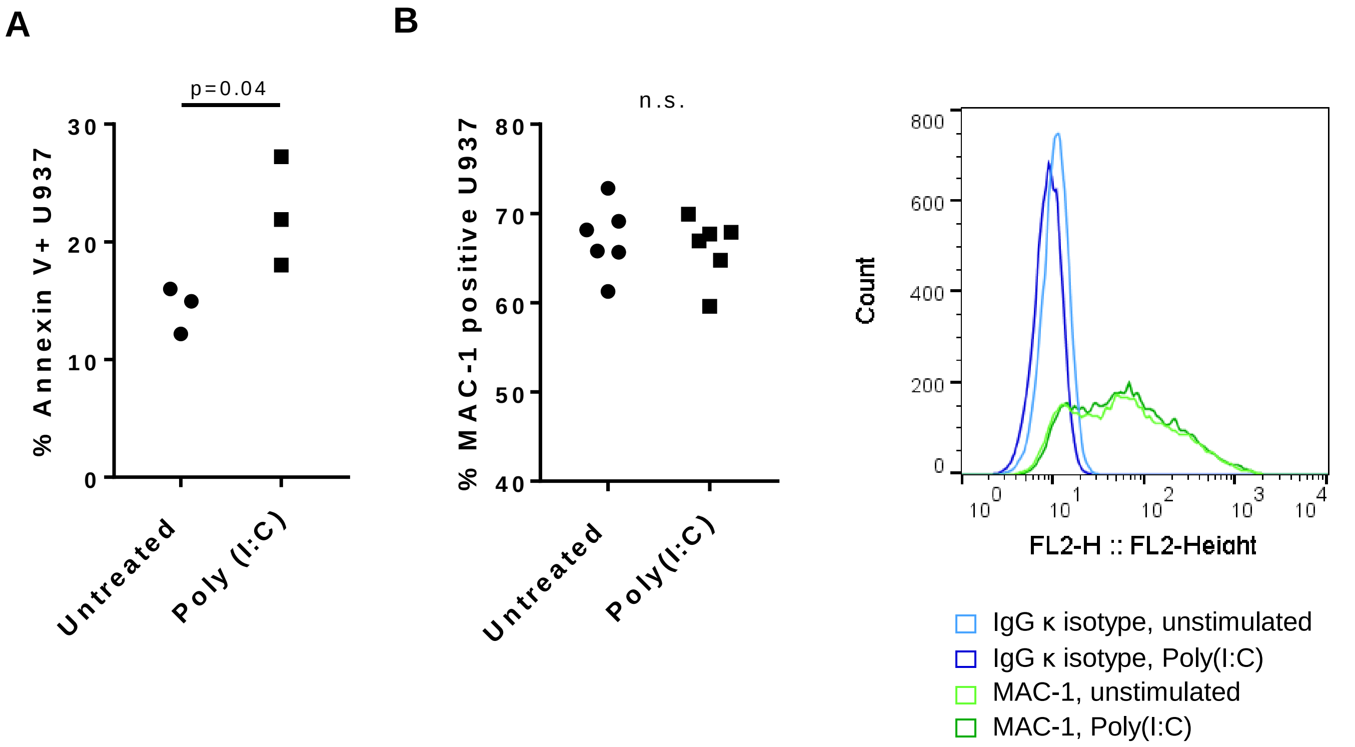

Supplementary Figure 1. Flow cytometry analysis of apoptosis and MAC-1 expression in U937 cells. (**A**) Percentage of apoptotic Annexin V-positive U937 cells upon stimulation with Poly(I:C) for 16h, shown are biological replicates. (**B**) Expression of MAC-1 on the surface of U937 cells in the presence or absence of Poly(I:C) for 16h. Quantification and representative FL2 histograms of MAC-1 expression in U937 cells. Statistics: (**A, B**) two tailed paired t test. n.s. = not significant.

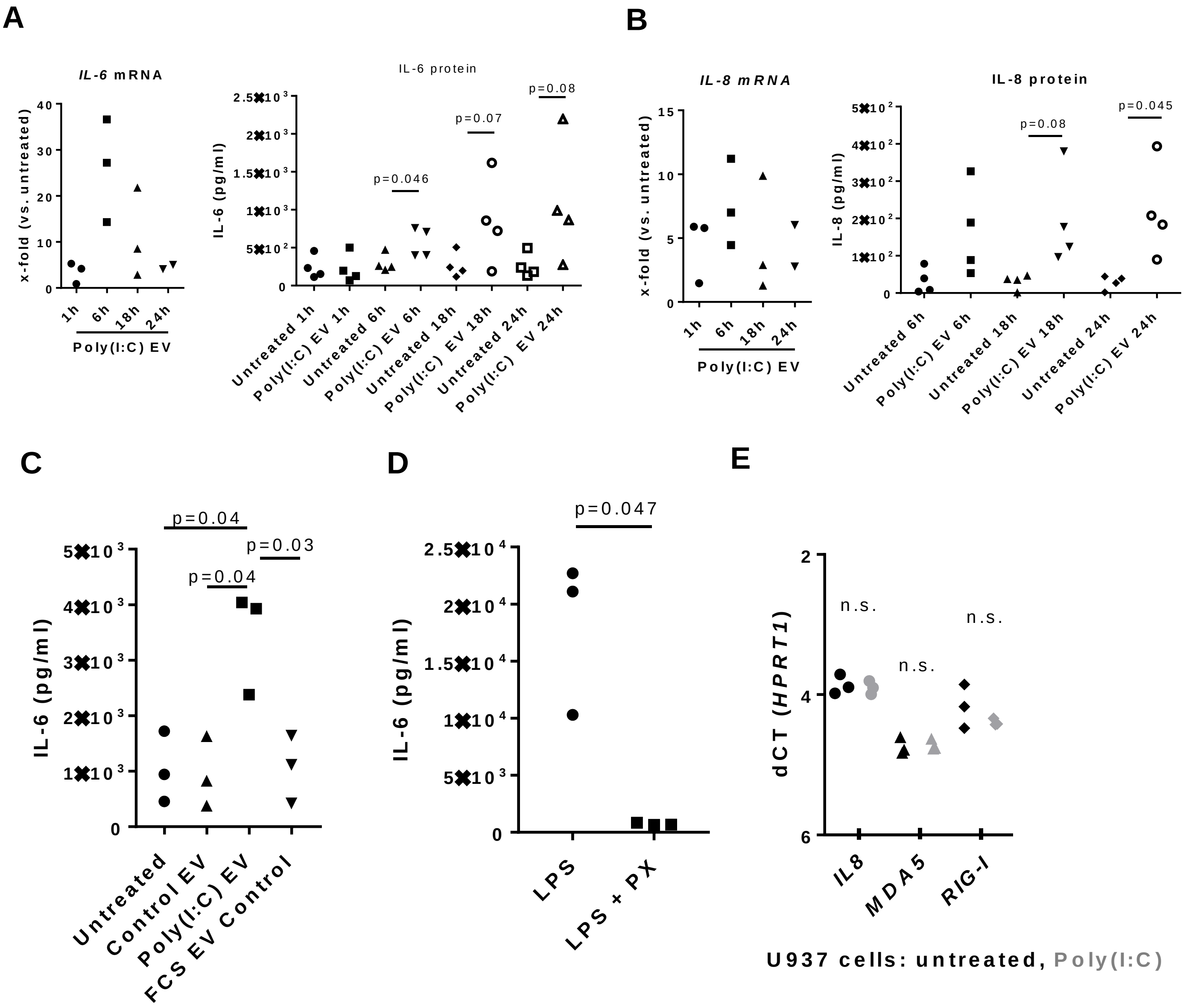

Supplementary Figure 2. Analysis of gene and protein expression in synovial fibroblasts (SFs) and U937 cells. (**A, B**) Time-dependent production of *IL-6* and *IL-8* mRNAs as well as IL-6 and IL-8 proteins in SFs treated with extracellular vesicles (EV) from Poly(I:C)-stimulated U937 cells [Poly(I:C) EV], as measured by qPCR and ELISA, shown are biological replicates. qPCR data: x-fold change compared to untreated SFs with normalization to 18S rRNA. (**C, D**) The production of IL-6 in SFs treated with (**C**) U937 cell-derived EV or fetal calf serum EV control (FCS EV) and (**D**) LPS ± Polymyxin B (PX), as measured by ELISA, shown are biological replicates. FCS EV control was used to control for the effects of potential residual FCS-derived EV. (**E**) The expression of pro-inflammatory and anti-viral genes in U937 cells treated or not with 20µg/ml Poly(I:C) for 16h, qPCR data shown as ΔCt with normalization to *HPRT1*, shown are biological replicates. Statistics: (**A** - ELISA, **B** - ELISA, **D**, **E**) two tailed paired t-test, (**C**) one-way ANOVA with Tukey's multiple comparisons test. n.s. = not significant.

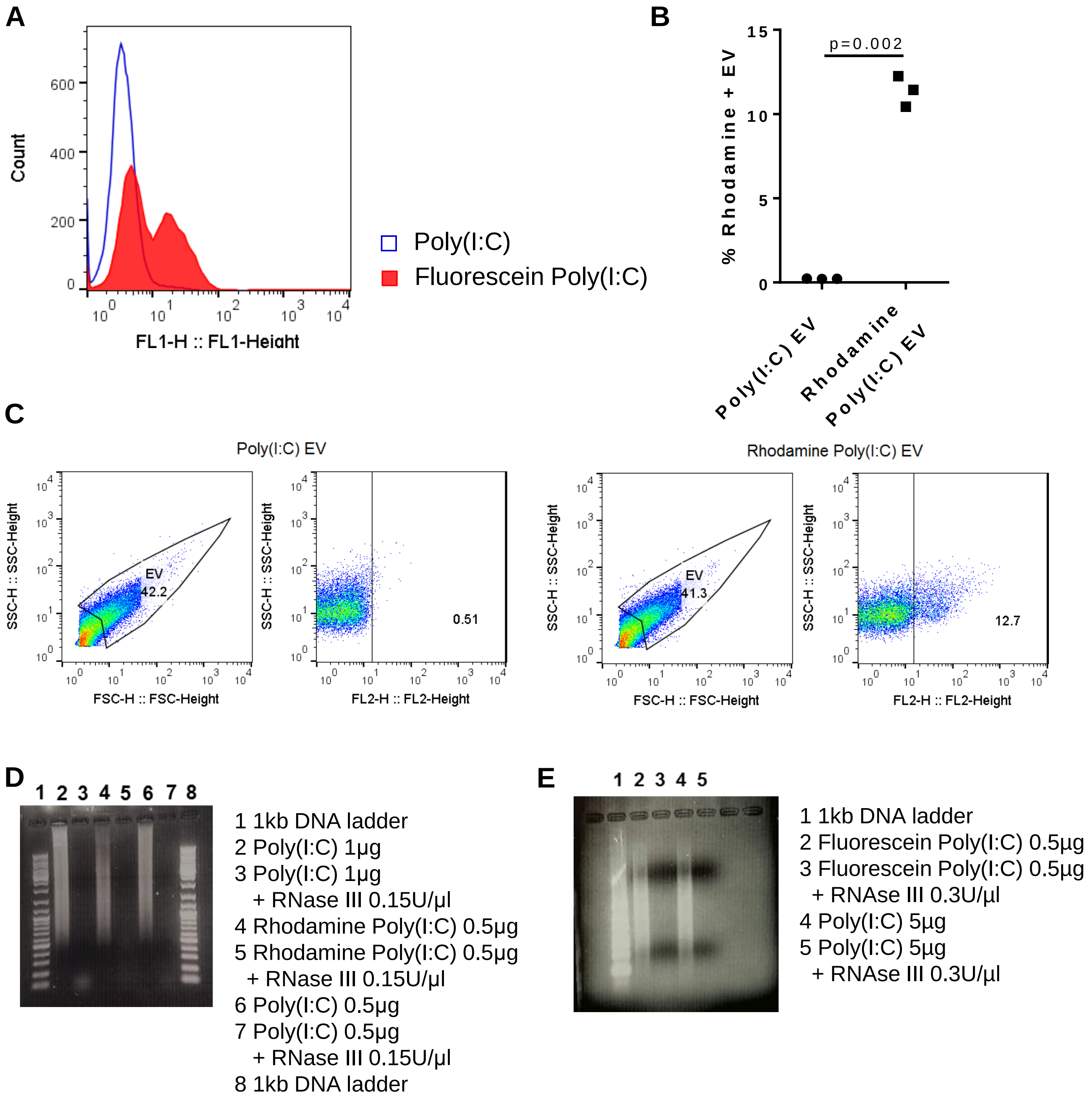

Supplementary Figure 3. Poly(I:C) associates with U937 cells and U937 cell-derived extracellular vesicles (EV). (**A**) Detection of Fluorescein Poly(I:C) on flow cytometer in U937 cells treated with Fluorescein Poly(I:C) or Poly(I:C). (**B**, **C**) Rhodamine Poly(I:C) detected on flow cytometer in EV derived from Poly(I:C)-stimulated or Rhodamine Poly(I:C)-stimulated U937 cells [Poly(I:C) EV and Rhodamine Poly(I:C) EV, respectively], shown are biological replicates. (**B**) Quantification of flow cytometry data and (**C**) representative SSC-H/FSC-H and SSC-H/FL2 flow cytometry charts (with % of Rhodamine + events) of EV. (**D, E**) Agarose gel electrophoresis of Poly(I:C), Rhodamine Poly(I:C) and Fluorescein Poly(I:C) digested or not with RNase III. Statistics: (**B**) two-tailed paired t test.

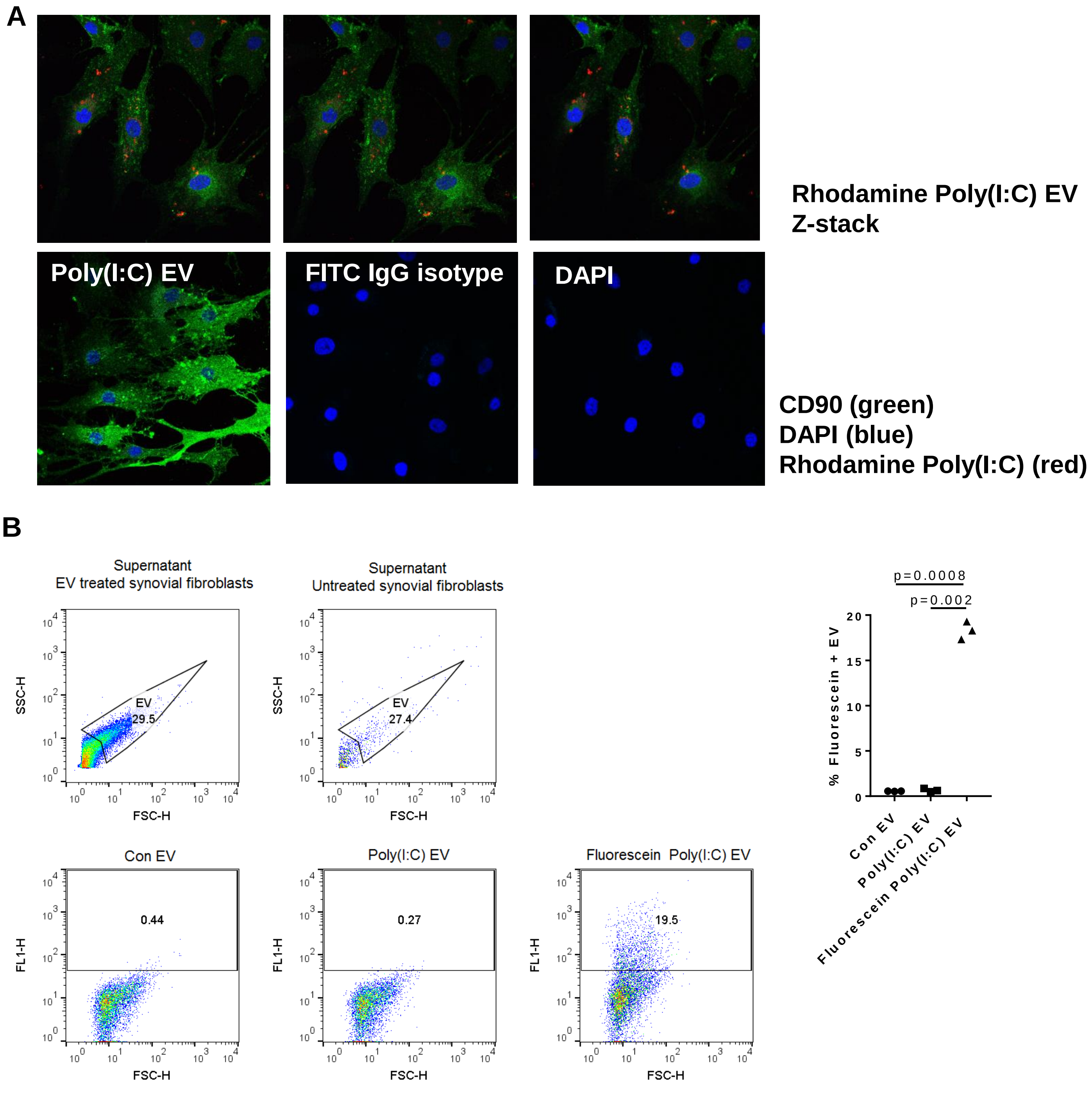

Supplementary Figure 4. U937 cell-derived extracellular vesicles (EV) shuttle Poly(I:C) to synovial fibroblasts (SFs). (**A**) Confocal microscopy of SFs treated for 24h with EV released from Rhodamine Poly(I:C)-stimulated U937 cells (3 sequential z-stack images) or Poly(I:C)-stimulated U937 cells [Rhodamine Poly(I:C) EV and Poly(I:C) EV, respectively]. Controls: SFs incubated with IgG isotype + DAPI or DAPI only (DAPI). Nuclei - DAPI (blue), CD90 (green, a fibroblast marker), Rhodamine Poly(I:C) (red). Magnification: 40x. (**B**) Fluorescein Poly(I:C) EV in supernatants of SFs treated with U937-cell derived Fluorescein Poly(I:C) EV, Poly(I:C) EV and control EV (Con EV, derived from unstimulated U937 cells) for 24h, as measured by flow cytometry. As a control , EV isolated from superntants of untreated SFs are shown. Representative SSC-H/FSC-H and FL1-H/FSC-H flow cytometry charts with % of fluorescein-positive events and quantification of flow cytometry data. Statistics: (**B**) one-way ANOVA with Tukey’s multiple comparisons test.

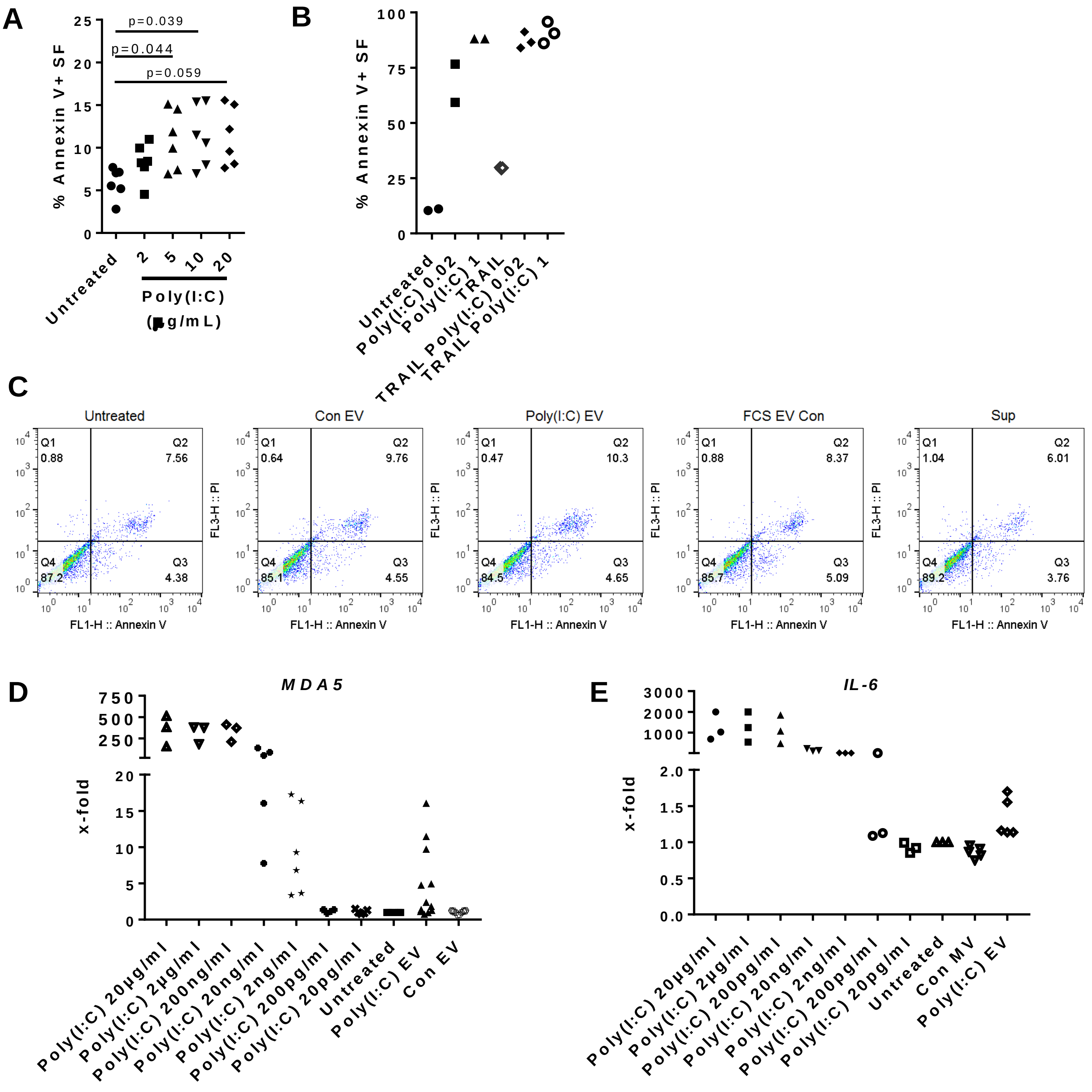

Supplementary Figure 5. Analysis of apoptotic, anti-viral and pro-inflammatory responses in synovial fibroblasts (SFs). Percentage of Annexin V-positive SFs, as measured by flow cytometry, upon (**A**) treatment with Poly(I:C) or (**B**) transfection of Poly(I:C) ± TRAIL, shown are biological replicates. (**C**) Representative flow cytometry charts of propidium iodide (FL3)/Annexin V (FL1) staining of SFs with % of cells in each quadrant. SFs were untreated or were treated with extracellular vesicles derived from unstimulated [Con EV] or Poly(I:C)-stimulated [Poly(I:C) EV] U937 cells, fetal calf serum (FCS) EV control or supernatants from the last washing step of Poly(I:C) EV pellets (Sup). FCS EV control and Sup were used to control for the effects of potential residual FCS-derived EV and the carryover of soluble Poly(I:C) and U937-derived mediators, respectively. Quantfication of data is presented in Fig.5A. (**D, E**) Induction of (**D**) *MDA5* and (**E**) *IL-6* mRNAs in SFs treated with Poly(I:C), Con EV or Poly(I:C) EV, shown are biological replicates. qPCR data normalized to *HPRT1* and shown as x-fold change vs. untreated SFs. Statistics: (**A**) one-way ANOVA with Tukey's multiple comparisons test and Geisser-Greenberg correction for unequal variances.

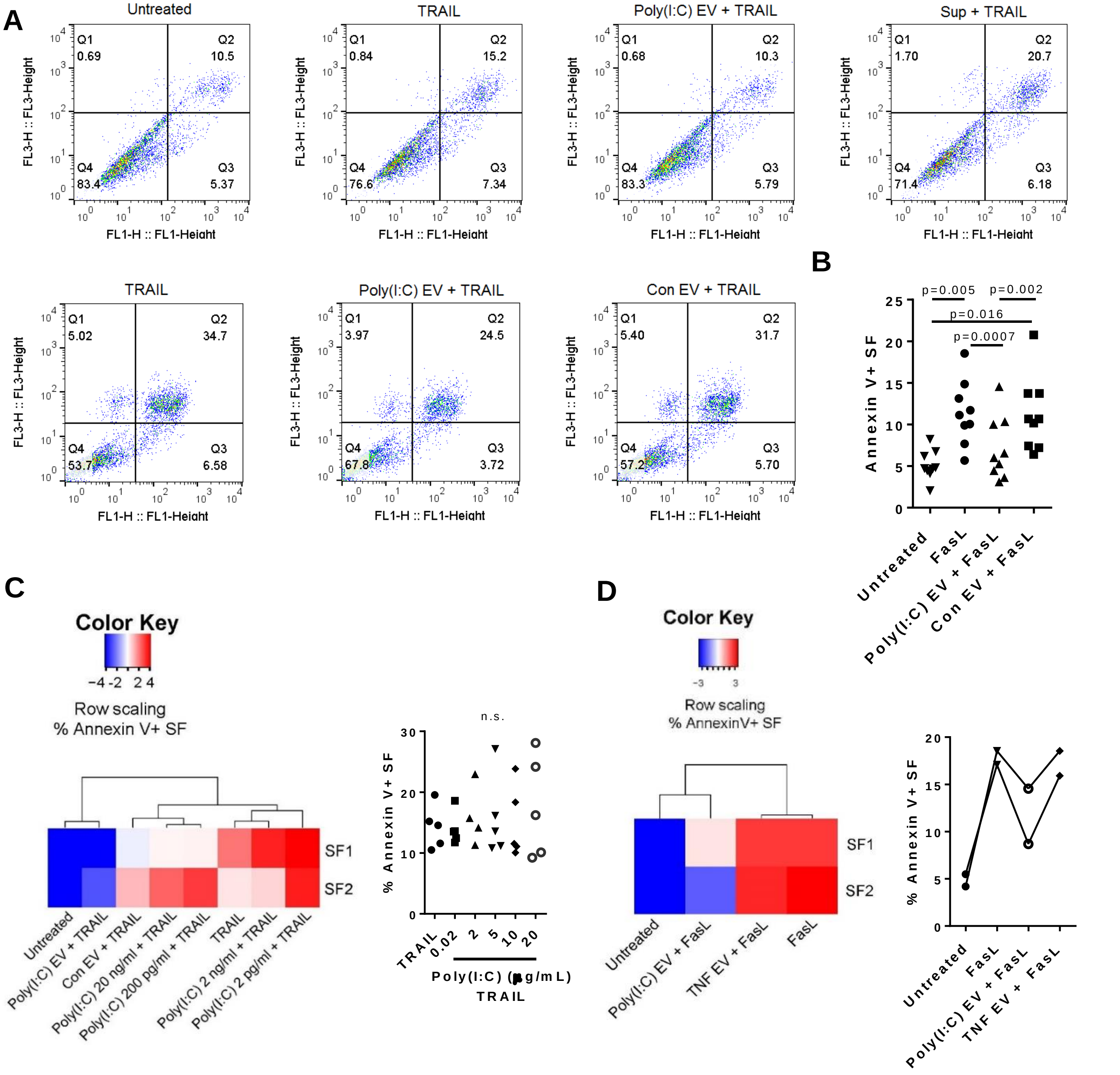

Supplementary Figure 6. Analysis of apoptotic responses in synovial fibroblasts (SFs) (**A**) Representative flow cytometry charts of propidium iodide (FL3)/Annexin V (FL1) staining of SFs with % of cells in each quadrant. SFs were untreated or were treated with TRAIL in the presence of extracellular vesicles derived from unstimulated [Con EV] or Poly(I:C)-stimulated [Poly(I:C) EV] U937 cells, or with supernatants from the last washing step of Poly(I:C) EV pellets (Sup). Sup were used to control for the carryover of soluble Poly(I:C) and U937-derived mediators, respectively. Quantfication of data is presented in Fig. 5C. (**B-D**) Percentage of Annexin V-positive SFs, as measured by flow cytometry, upon treatment with (**B**) Fas ligand (FasL) ± EV; (**C**) Poly(I:C) + TRAIL, (**D**) FasL ± Poly(I:C) EV or TNF EV (from TNF-stimulated U937 cells), shown are biological replicates. Heatmaps (based on flow cytometry analysis of Annexin V) show clustering of apoptotic responses in SFs undergoing (**C**) TRAIL-induced apoptosis or (**D**) FasL-induced apoptosis under specified experimental conditions. Statistics: (**B**, **C**) one-way ANOVA with Tukey's multiple comparisons test and (**C**) Geisser-Greenberg correction for unequal variances. n.s. = not significant.

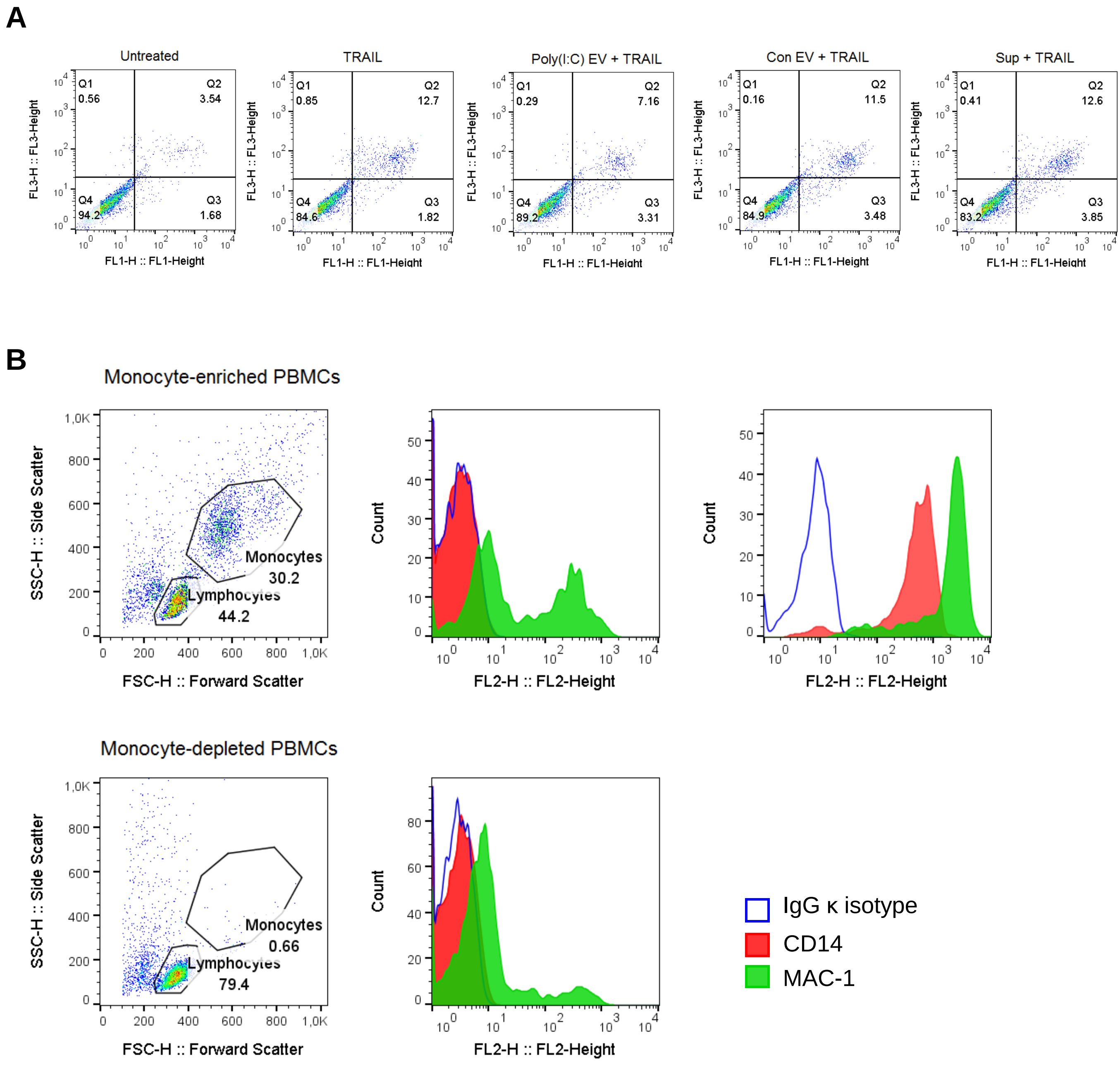

Supplementary Figure 7. Analysis of apoptotic responses in synovial fibroblasts (SFs) co-cultured with extracellular vesicles (EV) derived from peripheral blood mononuclear cells (PBMCs). (**A**) Representative flow cytometry charts of propidium iodide (FL3)/Annexin V (FL1) staining of SFs with % of cells in each quadrant. SFs were untreated or were treated with TRAIL in the presence of EV derived from unstimulated [Con EV] or Poly(I:C)-stimulated [Poly(I:C) EV] PBMCs, or with supernatants from the last washing step of Poly(I:C) EV pellets (Sup). Sup were used to control for the carryover of soluble Poly(I:C) or PBMC-derived mediators, respectively. Quantfication of data is presented in Fig. 5D. (**B**) Analysis of MAC-1 and CD14 expression on the surface of monocytes and lymphocytes using flow cytometry in the monocyte-enriched and the monocyte-depleted PBMC fractions isolated from a healthy donor.

A

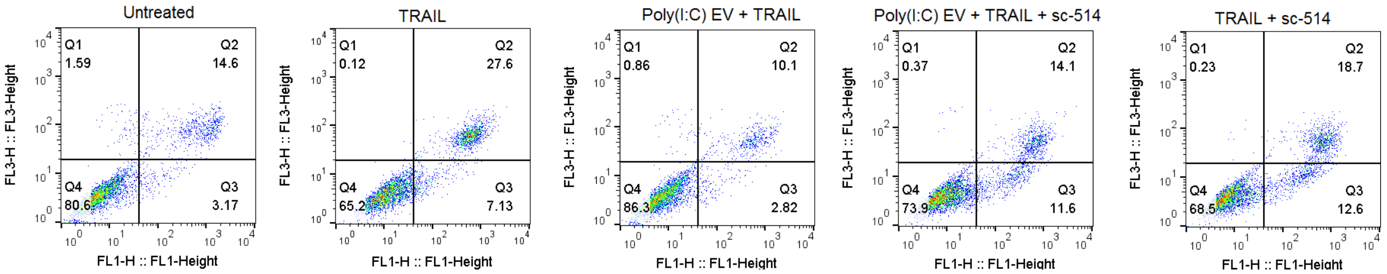

B

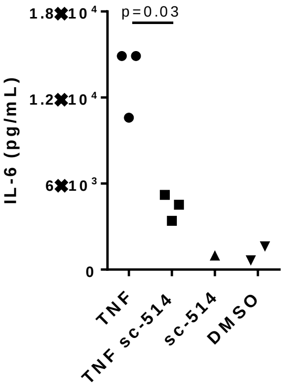

Supplementary Figure 8. Analysis of the role of NF-κB in pro-survival actions of U937 cell-derived extracellular vesicles (EV) during TRAIL-induced apoptosis of synovial fibroblasts (SFs). (**A**) Representative flow cytometry charts of propidium iodide (FL3)/Annexin V (FL1) staining of SFs with % of cells in each quadrant. SFs were untreated or were treated with TRAIL ± EV derived from Poly(I:C)-stimulated U937 cells [Poly(I:C) EV] in the presence or absence of sc-514, (50μM), the inhibitor of IKK-2. Quantfication of data is presented in Fig. 5G. (**B**) The production of IL-6 in SFs treated with TNF ± sc-514 (50μ*M*) or DMSO (control), shown are biological replicates. Statistics: (**B**) two tailed paired t-test.

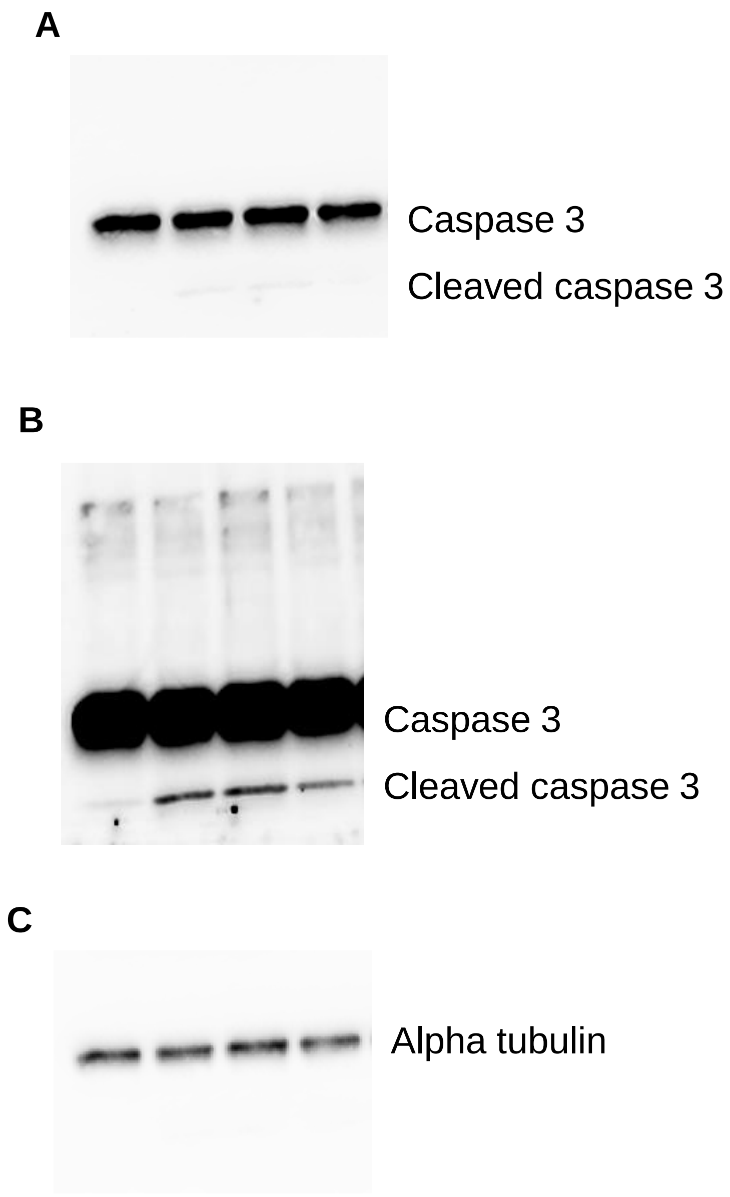

Supplementary Figure 9. Original Western blot images of (**A**) uncleaved caspase 3, (**B**) cleaved caspase 3 and (**C**) alpha tubulin, that are shown in Fig. 5B as merged image. For detection of different proteins, the nitrocellulose membrane from same gel was exposed to light for different periods of time. Rabbit anti-Caspase 3 antibody (Cell Signalling, #9662) detected (**A**) uncleaved caspase 3 after short exposure times (few sec), whereas (**B**) cleaved caspase 3 was detected at longer exposure times (few min; at this time uncleaved caspase 3 bands were overexposed). To quantify the bands of uncleaved caspase 3, short exposure times were used. (**C**) Alpha tubulin was detected on a sequential day on the same memebrane by using mouse anti α-tubulin antibodies (Abcam, #ab7291).

Supplementary Table 1
Sybr Green and TaqMan Primers

Gene	Sequence
HPRT1	F: 5'ATG GAC AGG ACT GAA CGT CTT G 3'<br>R: 5'GGC TAC AAT GTG ATG GCC TC 3'
GAPDH	F: 5' TCT CGC TCC TGG AAG ATG GT 3'<br>R: 5' GGG AAG CTT GTC ATC AAT GGA
TLR3	F: 5' CCT GGT TTG TTA ATT GGA TTA ACG A 3'<br>R: 5' TGAGGTGGAGTGTTGCAAAGG 5'<br>Probe: 5' ACCCATACCAACATCCCTGAGCTGTCAA 3'
MDA5	F: 5' ACA TTG CCA AGG ATC ACT TAG AC 3'<br>R: 5' AAC TAG CAG TAC CTT ATT GAC AAG 3'
RIG-I	F: 5' TTG AAA GAC ATG GGT ATA GAG TTA C '3<br>R: 5' AAT CTG TTC CAC TGG GAC ATT C '3
IL-6	F: 5' CCC TGA GAA AGG AGA CAT GTA AC 3'<br>R: 5' CCT CTT TGC TGC TTT CAC ACA TG 3'
IL-8	F: 5' TTG GCA GCC TTC CTG ATT TC 3'<br>R: 5' TGG CAA AAC TGC ACC TTC AC 3'
